# The French Pregnancy Cohort: Medication use during pregnancy in the French population

**DOI:** 10.1371/journal.pone.0219095

**Published:** 2019-07-17

**Authors:** Anick Bérard, Fatima Abbas-Chorfa, Behrouz Kassai, Thierry Vial, Kim An Nguyen, Odile Sheehy, Anne-Marie Schott

**Affiliations:** 1 Research Center, CHU Sainte-Justine, Montreal, Quebec, Canada; 2 Faculty of Pharmacy, University of Montreal, Montreal, Quebec, Canada; 3 EPICIME-CIC 1407 Lyon, Inserm, Pharmacotoxicology Department, CHU-Lyon, Bron, France; 4 University of Lyon 1, Lyon, France; 5 Laboratoire de Biométrie et Biologie Evolutive, University of Lyon 1, CNRS, UMR 5558, Villeurbanne, France; 6 Hospices Civils de Lyon, Service de biostatistique-bioinformatique, Pôle Santé Publique, Lyon, France; 7 Service Hospitalo-Universitaire de Pharmacotoxicologie, CHU-Lyon, Lyon, France; 8 Hospices Civils de Lyon, Pôle de Santé Publique, Lyon, France; 9 Université Lyon Claude Bernard Lyon 1, HeSPeR, Lyon, France; University of Oslo, NORWAY

## Abstract

**Purpose:**

We described the medication use during pregnancy in the French population using the French Pregnancy Cohort (FPC).

**Methods:**

The FPC was built with the sampling of all pregnant women included in the French Echantillon généraliste des bénéficiaires (EGB), which is a 1/97^th^ representative sample of the population covered by the French health insurance. The EGB includes anonymized information on the socio-demographic and medical characteristics of beneficiaries, and the health care services they have received such as diagnoses and procedure codes as well as data on filled reimbursed medication; EGB also includes data on hospital stays in all public and private French health facilities. Each filled prescription record contains information on drug brand and generic names, date of prescription and date of dispensing, quantity dispensed, mode of administration, duration of prescription, dosage, and prescribing physician specialty. FPC includes data on all pregnancies of women in the EGB (2010–2013). Date of entry in the FPC is the first day of pregnancy regardless of pregnancy outcome (spontaneous abortions or planned abortions (with or without medical reasons), deliveries), and data on women are collected retrospectively for a period of one year before pregnancy, and prospectively during pregnancy, and up to one year after delivery. The prevalence of prescribed medications before, during and after pregnancy was compared; comparison was also done between trimesters. Pregnancy outcomes are described and include spontaneous and planned abortions, livebirths, and stillbirths.

**Results:**

FPC includes data on 36,065 pregnancies. Among them, 27,253 (75.6%) resulted in a delivery including 201 stillbirths (0.7%). The total number of spontaneous abortions was 6,718 (18.6%), and planned abortions 2,094 (5.8%). The prevalence of filled medication use was 91.1%, 89.9%, and 95.6% before, during and after pregnancy, respectively. Although there was a statistically significant decrease in the proportion of use once the pregnancy was diagnosed (first trimester exposure, 76.4% vs. exposure in the year prior to pregnancy, 91.1% (p < .01)), post-pregnancy medication use was above the pre-pregnancy level (95.6%). Maternal depression was the most prevalent comorbidity during pregnancy (20%), and post-partum depression was higher in those who delivered a stillborn infant (38.8%) as well as in those with a spontaneous (19.5%) or planned abortion (22.4%) compared to those with a liveborn (12.0%).

**Conclusion:**

FPC is an excellent tool for the study of the risk and benefit of drug use during the perinatal period. FPC has the advantage of including a representative sample of French pregnant women, and study medications only available in France in addition to others available worldwide.

## Introduction

Evidence suggests that over 75% of women take at least one medication during pregnancy [1]. Much of this exposure occurs because more than half of pregnancies are unplanned [2], resulting in millions of fetuses being exposed to medications during organogenesis because women did not know they were pregnant. Additional exposures to medications also occur during planned pregnancies, either due to maternal chronic illnesses, or acute conditions that develop during pregnancy. Yet, data on the safety of medications used during pregnancy (for both women and fetuses) is generally lacking.

Currently, observational studies on the risks and potential benefits of medication use during pregnancy are the only way to close the knowledge gap, but majority of these studies focus on the health of the fetus/child, and fail to look specifically at the health of the pregnant women. The latter is key to any decision on medication use during pregnancy–whether it is on use versus non-use of drug or which therapy is chosen–as potential maternal health benefits as well potential harms and benefits to the fetus are important considerations. Indeed, while data on fetal outcomes are important for clinicians and mothers when making treatment decisions, lack of information on maternal physical and mental health outcomes precludes overall understanding of the risks and benefits of prescription medication use during pregnancy.

Data from the population-based Quebec Pregnancy Cohort (QPC) has shown that 56% of pregnant women have an on-going medication prescription during gestation [3]. Other estimates have been calculated in The Netherlands (86%) [4], and Norway (61%) [5]. In France however, surveys have suggested that 74%-100% of pregnant women take medications during gestation [6–9].

The single-payer health care in France has facilitated the linking of inpatient, outpatient (including emergency department (ED) visits), hospital, ambulatory care, and pharmaceutical data [10–12]. France has made available a representative sample of its population’s health care data as well as data on medication use and hospitalisations to researchers working in non-profit organisations (Échantillon Généraliste des Bénéficiaires (EGB)) [10–12]. Demailly *et al*. [13], using data from the EGB, found that over 70% of pregnant women were exposed to medications during gestation. Given that the majority of medication use during gestation are from inadvertent exposure [14] because women do not know they are pregnant, Demailly *et al*.’s [13] estimate is likely an underestimation since they did not consider filled prescriptions before pregnancy with duration overlapping the beginning of pregnancy; they also excluded many pregnancies because they were not able to determine either the beginning or the end of pregnancy, which can potentially lead to bias.

Given the limited information on the use of medications during pregnancy at a population level in France; the fact that some medications, such as antidepressants, benzodiazepines and vitamins, are more widely used in France than in other countries; and the fact that some medications are only marketed/used in the French population, it is essential to fully assess prevalence, and trends of drug use during pregnancy in order to have a significant impact on short- and long-term outcomes in this population. In other countries, it has been reported that women using medications during gestation are at an increased risk of induced/planned and spontaneous abortions, prematurity, multiplicity, and post-partum depression [3–5]. Determinants of medication use at the beginning of pregnancy include older maternal age, being on welfare, visiting multiple physicians, and having a history of diabetes, hypertension, depression or asthma [3]. Given differences between maternal characteristics between countries, a French pregnancy cohort could be helpful to primary caregivers who are ultimately making the decision to prescribe or not prescribe medication during gestation. Because access and delivery of health care vary between countries, the FPC was established to study short- and long- term effects of medication use during gestation on mothers. With this paper, we aim to present the FPC as well as the definitions developed to identify pregnancies and pregnancy outcomes within the EGB, and provide information on the prevalence of filled prescriptions during the perinatal period. In addition, we aim to provide baseline population-based results in order to highlight the FPC’ potential for perinatal pharmacoepidemiologic research.

## Methods

### Ethics statement

The use of EGB was done by an authorized biostatistician (FAC). The CHU de Lyon’s Ethics’ Committee approved the formation of the FPC and its analyses.

### The French Pregnancy Cohort (FPC)

FPC is a representative sample of all French pregnant women with longitudinal follow-up data.

The *Echantillon généraliste des bénéficiaires* (EGB) is a 1/97^th^ representative sample of the population covered by the French health insurance and includes data from two administrative databases, the *SNIIRAM (reimbursement claims in ambulatory care)*, and the *Programme de Médicalisation des Systèmes Informatiques* (PMSI, national hospital discharge summaries) [15]. It contains anonymous information on the socio-demographic and medical characteristics of beneficiaries, and the health care services they have received such as diagnoses and procedure codes, data on filled reimbursed medications, and specialty of the prescribers. Each prescription record contains information on drug brand and generic names, date of prescription and date of dispensing, quantity dispensed, mode of administration, duration of prescription, dosage, and prescribing physician specialty. All drugs are classified according to their drug identification number (DIN) code, and the Anatomical Therapeutic Chemical (ATC) code. Over-the-counter (OTC) drugs and vitamins are not included in the EGB database if they are not prescribed or reimbursed; drugs administered during hospitalizations are also not included. Data on medications recorded in the EGB are based on prospective fillings. 1/97^th^ of people insured by the French social security were randomly sampled and form the EGB. The number of people protected by the general scheme in the EGB was 46,891,934 in 2008, and breakdown per age and sex is very similar to that of the overall French population [15].

The *Programme de Médicalisation des Systèmes Informatiques* (PMSI) included in EGB includes data on short stays in public and private French health facilities since 1996. It is a national database where each patient’s stay is documented with personal information (age, sex, postal code of the area of residence), health medical data (diagnostic code according to the international classification of diseases, 10^th^ edition (ICD-10), medical procedure codes according to the French ‘Classification des Actes Médicaux’, and medical department of stay).

For each calendar year studied, using data from the PMSI, an extraction of deliveries, spontaneous or induced/planned abortions was performed using an algorithm developed specifically for the FPC, which used data from medical and hospital visits/stays and procedures as well as diagnostic codes (ICD-10 codes) (**Appendix A in S1 File**). Hence, from the medical and hospitalisation visits data, pregnancies that were registered to the health insurance were identified and extracted using date of beginning of pregnancy listed in the standardized declaration of pregnancy form ('first prenatal medical examination'). These extractions constitute a set of pregnancies for which the ultrasound was performed in the first, second or third trimester of pregnancy. Gestational age, the date of the declaration of pregnancy, dates of the ultrasounds, and date of the pregnancy outcome (delivery, spontaneous or induced/planned abortions) were used for the estimation of the date of the beginning and end of pregnancy as well as the dates of the three trimesters. Although minimal compared to deliveries performed in hospital settings, data on deliveries occurring outside hospitals were also identified. The medicated induced/planned abortions were identified using the CCAM code JNP001 or with a procedure for evacuation of a pregnant uterus in the first trimester (CCAM code JNJD002) and from the benefits codes (1981, 2411, 2419, 2422, 2423, 2425, 3329) (**Appendix B in S1 File**). As gestational age is not recoverable for these city data, it was arbitrarily set at 41 weeks for city deliveries and 7 for medicated induced/planned abortions. This pregnancy selection algorithm can be reapplied to recent years if updates are made to the CCAM procedure and act codes, ICD-10 diagnosis codes or insurance benefits.

To be included in the FPC, women had to have at least one procedure related to the ‘*Système National d'Informations Inter Régimes de l'Assurance Maladie* (SNIIRAM)’ procedure codes, acts or diagnosis PMSI codes of pregnancy that began between 01/01/2010 and 31/12/2013 identified in the EGB. Women with multiple pregnancies were eligible. In addition of having a pregnancy, we required that women had complete health insurance coverage in the year before, during and the year after pregnancy in order to have complete data on diagnoses and medication fillings. The unit of analysis was a pregnancy. In the FPC, women were prospectively followed from at least the year before pregnancy, during pregnancy until the end of pregnancy (induced/planned or spontaneous abortion, or delivery, whichever occurs first), and at least up to 1-year post-partum. Women were treated and followed prospectively as part of the usual health care management in France before, during and after pregnancy. In France, once having a positive pregnancy test, the woman may be checked with the same gynecologist throughout her pregnancy, including 3 ultrasound scans (1^st^ at 12 weeks, 2^nd^ at 22–24 weeks, and 3^rd^ at 30–35 weeks of pregnancy, respectively) [16–19]. Specifically, from the EGB, we selected data on all the variables that characterized a pregnant woman (age, insurance, government financial support, geographic code) as well as the variables that described the physician visits (date in day/month/year, prescriber, diagnosis, procedure). As mentioned above, a visit was defined as a consultation for prenatal ultrasound, an obstetrical procedure, or any intervention performed by a midwife (including childbirth preparation). Ultrasound could be performed by a general practitioner (GP), a gynecologist, an obstetrician, an imaging center, or a midwife.

The FPC includes data on all pregnancies between 2010–2013 included in the EGB—data on 39,364 pregnant women.

### Baseline characteristics and prevalence of prescribed medication use before pregnancy and during the perinatal period

Baseline data on FPC are presented here for the study period 2010–2013. Characteristics of women were assessed on the first day of gestation (1DG); defined as the first day of the last menstrual period. Medication exposures included all filled reimbursed medications by the *Caisse National d’Assurance Maladie* (CNAM) obtained by prescription and dispensed by a pharmacist. Prevalences of prescribed medication exposures are presented according to the 3 following time-windows of interest: 1) before pregnancy (12 months before the 1DG), 2) during pregnancy (1DG until the end of pregnancy (spontaneous or planned abortions, or delivery)), and after pregnancy (12 months after the end of pregnancy). The pregnancy was also divided by trimesters. The first trimester was defined as the time from the 1DG until the 14^th^ completed week of gestation, the second trimester (between the15^th^ week and the 27^th^ completed week of gestation), and the third trimester (between the 28^th^ week until the end of the pregnancy). Exposure to prescribed medications was defined as having at least one prescription filled during the time-window of interest or one prescription filled before the beginning of the time-window but with duration overlapping the interval. The top 50 drugs per classes and types overall during pregnancy, and in each trimester are presented.

### Pregnancy outcomes

Pregnancy outcomes were evaluated at the end of the pregnancy. Only clinically detected spontaneous and induced/planned abortions are identified and reported here. Stillbirths were identified without specific causes; and the prevalence of multiplicity is also presented. Prematurity was defined as being born before the 37^th^ completed week of gestation among those with a delivery.

### Maternal chronic diseases and postpartum depression

The prevalence of the following maternal chronic conditions were considered, and measured in the year before and during pregnancy: hospitalisation for diabetes (ICD-10 codes E10-E14 and R730) or at least one reimbursed filled prescription for diabetes medications (ATC code A10); hypertension (ICD-10 codes I10-I15, O10-O16, or at least one prescription for antihypertensive drugs ATC code C02); and depression (ICD-10 'F31’, ‘F39’, ‘F41’, ‘F300’, ‘F301’, ‘F302’, ‘F308’, ‘F309’, ‘F320’, ‘F322’, ‘F323’, ‘F328’, ‘F329’, ‘F330''F331’, ‘F332’, ‘F333’, ‘F334’, ‘F338’, ‘F339’, ‘F341’, ‘F348’, ‘F349’, ‘F380’, ‘F381’, ‘F388’, ‘F431’, ‘F432’, ‘F438’, ‘F530' or at least one prescription for antidepressants (ATC codes N06A, N05B N05C)). Post-partum depression was defined as having a diagnosis of post-partum depression or depression (ICD-10 codes: 'F31’, ‘F39’, ‘F41’, ‘F300’, ‘F301’, ‘F302’, ‘F308’, ‘F309’, ‘F320’, ‘F322’, ‘F323’, ‘F328’, ‘F329’, ‘F330''F331’, ‘F332’, ‘F333’, ‘F334’, ‘F338’, ‘F339’, ‘F341’, ‘F348’, ‘F349’, ‘F380’, ‘F381’, ‘F388’, ‘F431’, ‘F432’, ‘F438’, ‘F530' or at least one prescription for antidepressants (ATC codes N06A, N05B N05C)) in the 12 months after giving birth, considering that post-partum depression can well be detected after the traditional 2 months post-delivery. Asthma was defined as having one diagnosis for asthma in the year before or during pregnancy (ICD-10 J42) or having filled at least one anti-asthma medications in the same time-period (ATC code R03).

### Statistical analyses

Characteristics of pregnant women are presented as proportions for categorical variables and means with standard deviations (SD) for continuous variables. Prevalences of exposure to prescribed medications are presented as proportions of pregnancies exposed for all prescribed medications combined and by class for each time-window interval. Prevalences of prescribed medications are compared between intervals using generalized equations models (GEE). Prevalences of pregnancy outcomes are also presented as proportions. All analyses were conducted using the SAS System V9.4 (SAS Institute Inc., North Carolina, USA).

## Results

### FPC characteristics and pregnancy outcomes

**Fig 1:**
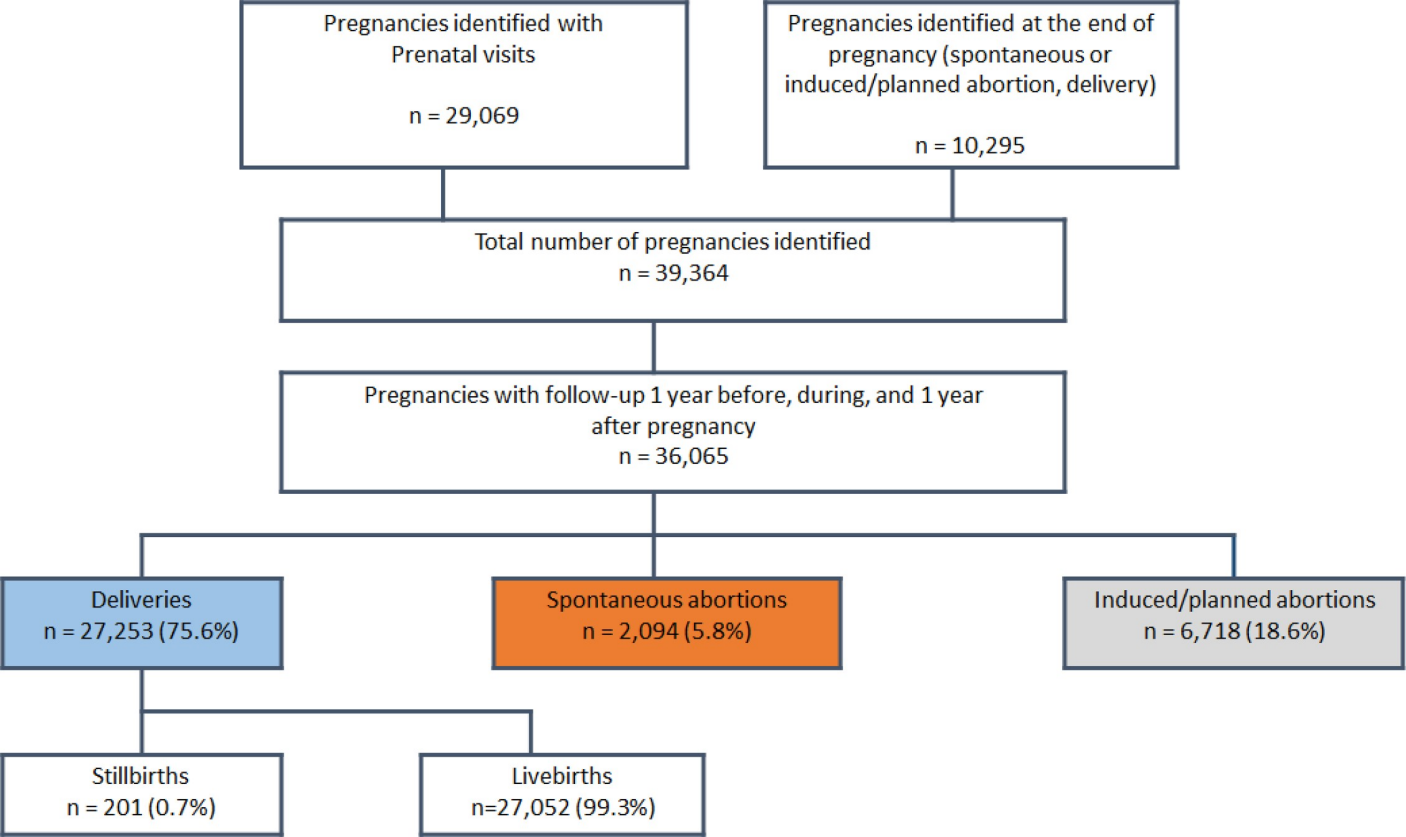
FPC–Selection of pregnancies–January 2010-December 2013.

**Fig 2:**
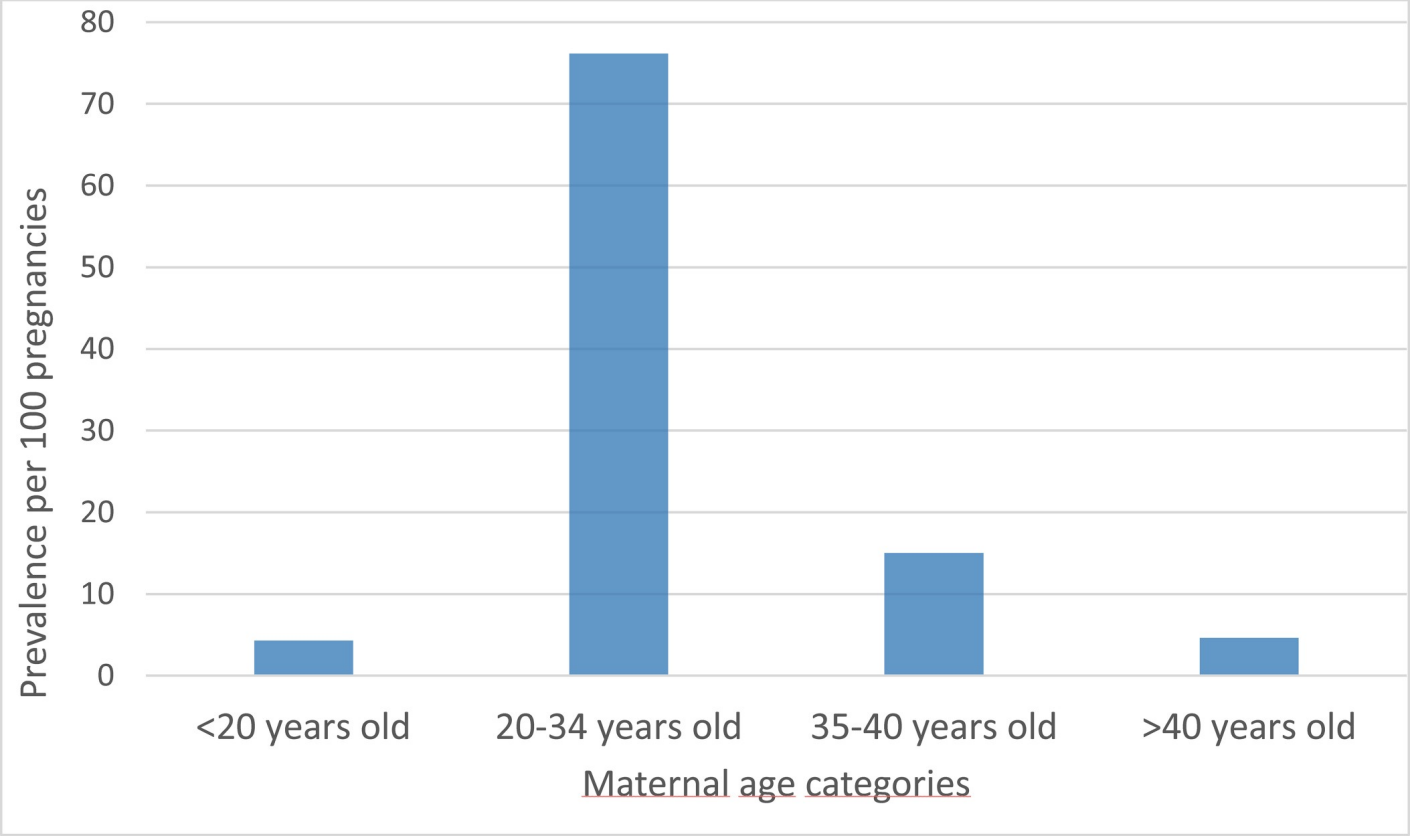
FPC–Pregnancy prevalence according to maternal age.

**Fig 3:**
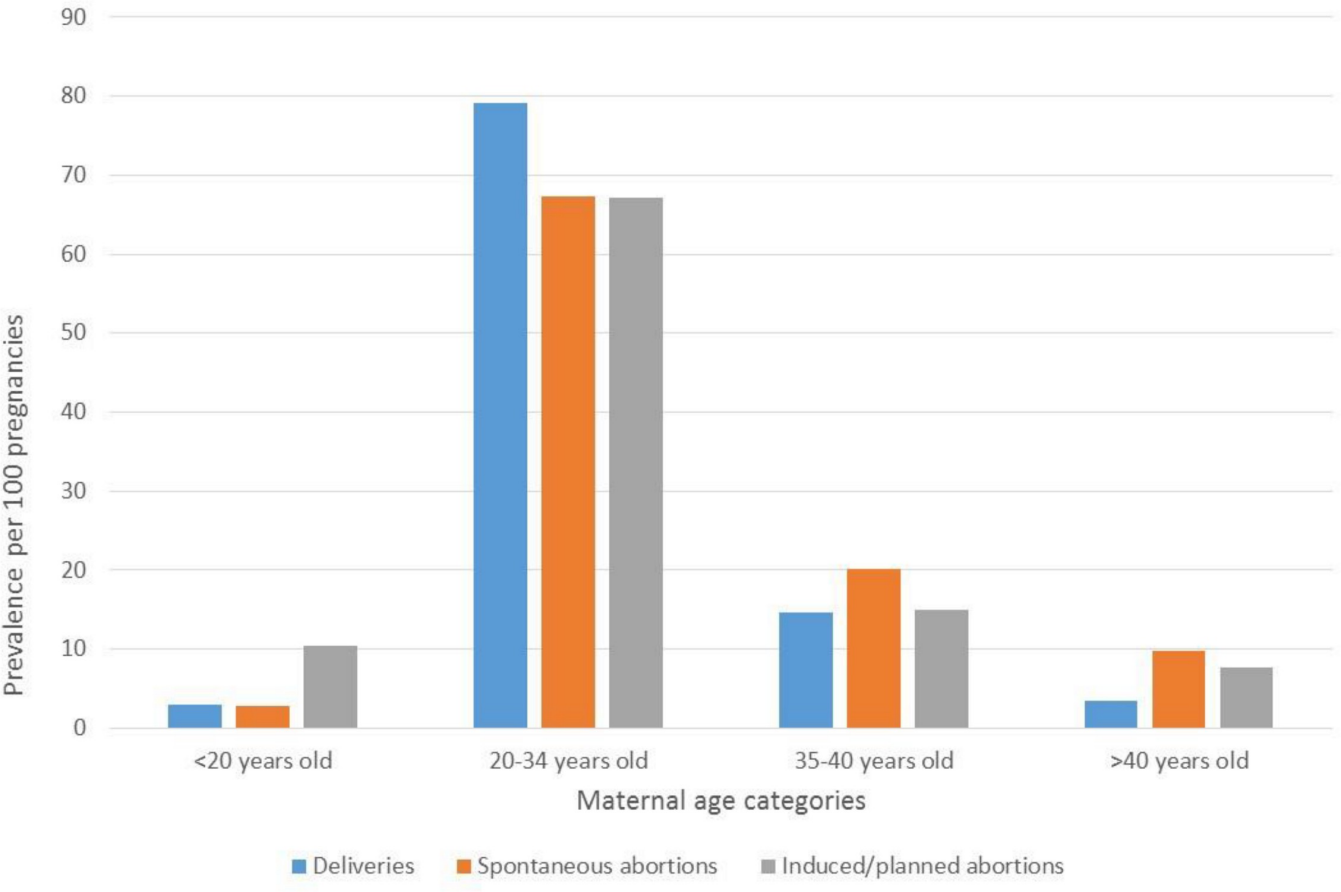
FPC–Prevalences according to maternal age and pregnancy outcome.

**Fig 1** summarizes the construction of the FPC. For the period 2010–2013, the FPC is comprised of 36,065 pregnancies with complete follow-up data in the year before, during, and the year after pregnancy. The majority of pregnancies resulted in deliveries (27,253 (75.6%)) including 201 stillbirths (0.7%); the remaining ended in spontaneous (2,094 (5.8%)) or induced/planned abortions (with or without medical reasons) (6,718 (18.6%)) (**Fig 1**). Pregnant women were mostly between 20–34 years old (76.2%) (**Fig 2**). Pregnancy outcomes varied according to maternal age (**Fig 3**); there were more planned abortions among those 20 years of age or younger, more deliveries among women aged 20–34 years, and more spontaneous abortions among women older than 35 years old, which is concordant with the literature [3].

**Table 1** describes the characteristics of pregnancies included in the FPC. Overall, the FPC includes data on equal numbers of pregnancies by calendar year of the cohort (approximately 25% in each of the 4 calendar years studied). Spontaneous and induced/planned abortions occurred at a mean gestational age (GA) of 9.9 weeks (standard-deviation (SD) 4.0) for spontaneous abortions, and 8.0 weeks (SD 2.5) for induced/planned abortions. The prevalence of prematurity among livebirths was 6.5% with the majority being near term births (32–37 weeks GA); the majority of stillbirths occurred between 22–28 weeks GA (49.8%). The majority of deliveries were singletons (98.1% (n, 24,248) in livebirths, and 93.4% (n, 170) for stillbirths).

**Table 1:** French Pregnancy Cohort (FPC)–Characteristics of the population– 2010–2013.

	All pregnancies 2010–2013 (n = 36,065)	Livebirths (n = 27,052)	Stillbirths (n = 201)	Spontaneous abortions (n = 2,094)	Induced/planned abortions (n = 6,718)
**Calendar year of start of pregnancy (denominator)**	36,065	27,052	201	2,094	6,718
2010	8,849 (24.5%)	6,626 (24.5%)	44 (21.9%)	494 (23.6%)	1,685 (25.1%)
2011	8,764 (24.3%)	6,597 (24.4%)	53 (26.4%)	459 (21.9%)	1,655 (24.6%)
2012	9,214 (25.5%)	6,933 (25.6%)	55 (27.4%)	580 (27.7%)	1,646 (24.5%)
2013	9,238 (25.6%)	6,896 (25.5%)	49 (24.4%)	561 (26.8%)	1,732 (25.8%)
**Maternal age (years) (denominator)**	36,065	27,052	201	2,094	6,718
Mean (SD)	29.4 (5.8)	29.5 (5.4)	29.9 (6.0)	31.1 (6.2)	28.6 (7.1)
<20	1,536 (4.3%)	780 (2.9%)	4 (2.0%)	56 (2.7%)	696 (10.4%)
20–34	27,464 (76.2%)	21,400 (79.1%)	148 (73.6%)	1,409 (67.3%)	4,507 (67.1%)
35–40	5,402 (15.0%)	3,943 (14.6%)	35 (17.4%)	423 (20.2%)	1,001 (14.9%)
>40	1,663 (4.6%)	929 (3.4%)	14 (7.0%)	206 (9.8%)	514 (7.7%)
**Gestational age (weeks) (denominator)**	29,935	26,908	201	565	2,261
Mean (SD)	36.1 (9.2)	39.0 (1.9)	29.6 (6.1)	9.9 (4.0)	8.0 (2.5)
< 22	2,827 (9.4%)	1 (0.0%)	0 (0.0%)	565 (100.0%)	2,261 (100.0%)
22 - <28	201 (0.7%)	101 (0.4%)	100 (49.8%)	0 (0.0%)	0 (0.0%)
28 - <32	170 (0.6%)	145 (0.5%)	25 (12.4%)	0 (0.0%)	0 (0.0%)
32 - <37	1,541 (5.1%)	1,506 (5.6%)	35 (17.4%)	0 (0.0%)	0 (0.0%)
≥37	25,196 (84.2%)	25,155 (93.5%)	41 (20.4%)	0 (0.0%)	0 (0.0%)
**Multiplicity (denominator)**	24,916	24,712	182	16	6
Singleton	24,428 (98.0%)	24,248 (98.1%)	170 (93.4%)	7 (43.8%)	3 (50.0%)
multiple (twins, triplets, etc.)	488 (2.0%)	464 (1.9%)	12 (6.6%)	9 (56.3%)	3 (50.0%)
**Maternal comorbidities in the year before and during pregnancy (denominator)**	36,065	27,052	201	2094	6,718
Depression	7,206 (20.0%)	5,183 (19.2%)	60 (29.9%)	429 (20.5%)	1,534 (22.8%)
Asthma	5,081 (14.1%)	4,021 (14.9%)	25 (12.4%)	258 (12.3%)	777 (11.6%)
Diabetes	2,200 (6.1%)	2,085 (7.7%)	11 (5.5%)	31 (1.5%)	73 (1.1%)
Hypertension	1,508 (4.2%)	1,419 (5.2%)	18 (9.0%)	21 (1.0%)	50 (0.7%)
**Post-partum depression in the year after the end of pregnancy**	5,247 (14.5%)	3,253 (12.0%)	78 (38.8%)	408 (19.5%)	1,508 (22.4%)
Medication use* **– 1 year before pregnancy (denominator)**	36,065	27,052	201	2,094	6,718
	32,839 (91.1%)	24,877 (92.0%)	189 (94.0%)	1,894 (90.4%)	5,879 (87.5%)
Mean (SD)	10.8 (9.6)	10.8 (9.4)	11.4 (10.0)	11.6 (10.0)	10.5 (10.1)
Medication use* **–during pregnancy (denominator)**	36,065	27,052	201	2,094	6,718
	32,408 (89.9%)	26,319 (97.3%)	194 (96.5%)	1,561 (74.5%)	4,334 (64.5%)
Mean (SD)	8.1 (6.5)	9.8 (6.3)	8.0 (5.3)	3.4 (3.7)	2.6 (3.1)
Medication use* **– 1 year after pregnancy (denominator)**	36,065	27,052	201	2,094	6,718
	344,75 (95.6%)	26,179 (96.8%)	197 (98.0%)	1,968 (94.0%)	6,131 (91.3%)
Mean (SD)	11.4 (9.0)	11.3 (8.6)	14.8 (10.0)	13.1 (10.2)	11.3 (10.1)
Medication use* **– 1st trimester (0–98 days) (denominator)**	36,065	27,052	201	2,094	6,718
	27,554 (76.4%)	21,524 (79.6%)	169 (84.1%)	1,553 (74.2%)	4,308 (64.1%)
Mean (SD)	3.5 (3.6)	3.7 (3.7)	4.1 (3.6)	3.3 (3.5)	2.6 (3.1)
Medication use* **– 2nd trimester (99–182 days) (denominator)**	27,387	27,047	201	83	56
	22,416 (81.8%)	22,180 (82.0%)	161 (80.1%)	43 (51.8%)	32 (57.1%)
Mean (SD)	3.4 (3.2)	3.4 (3.2)	3.3 (3.2)	1.8 (2.4)	1.6 (2.0)
Medication use* **– 3rd trimester (183-end) (denominator)**	27,014	26,919	95	0	0
	23,932 (88.6%)	23,861 (88.6%)	71 (74.7%)	NA	NA
Mean (SD)	4.1 (3.3)	4.1 (3.3)	2.8 (3.1)	NA	NA

Note: All estimations are presented as numbers and percentages unless stated otherwise.

The prevalences were not always calculated on the same number of subjects given that some variables had missing data. The denominators used are therefore presented.

*Medication refers to filled reimbursed medications.

SD, standard deviation; NA, not applicable.

### Maternal comorbidities in the FPC

**Fig 4:**
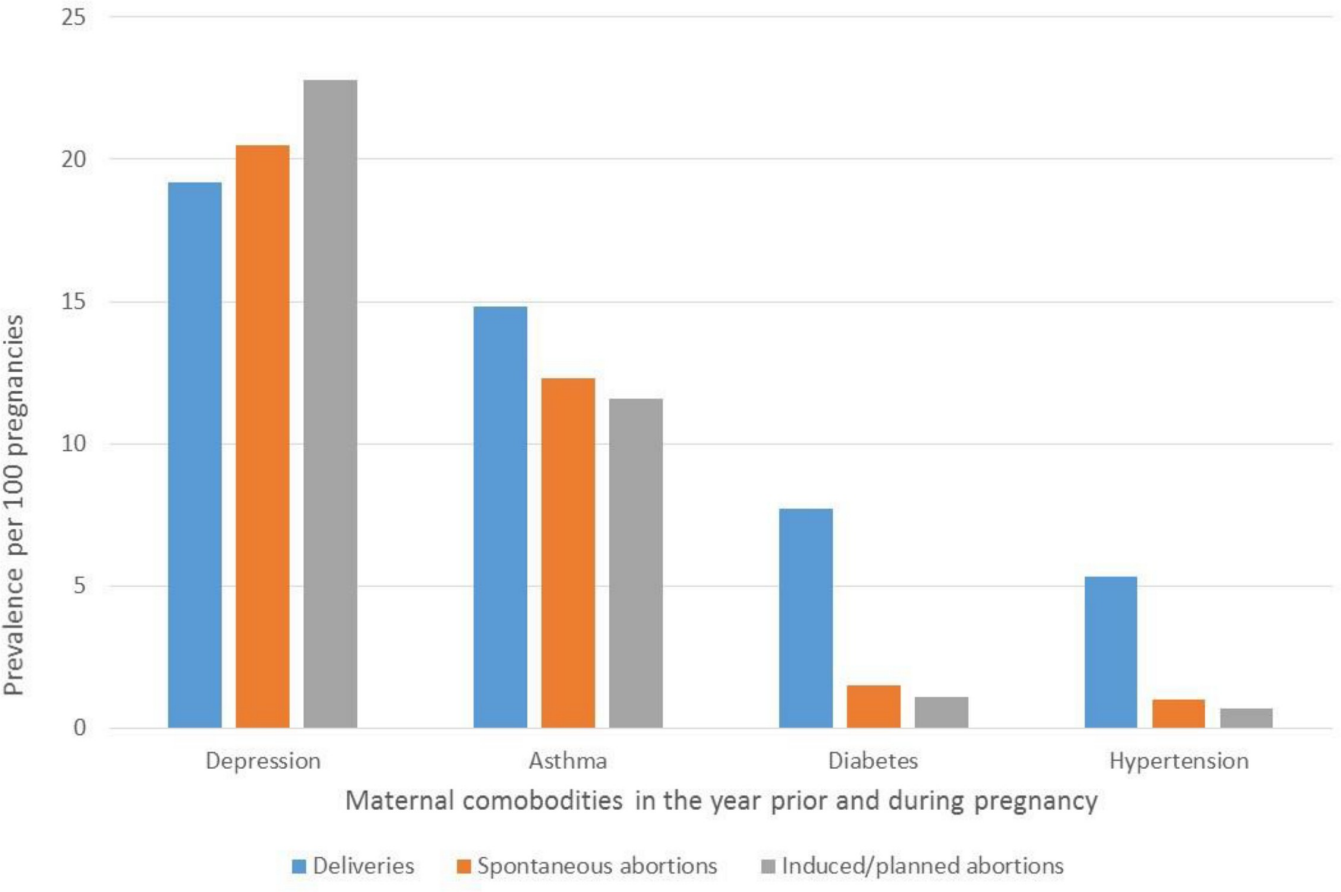
FPC–Prevalences of maternal comorbidities according to pregnancy outcome.

**Fig 5:**
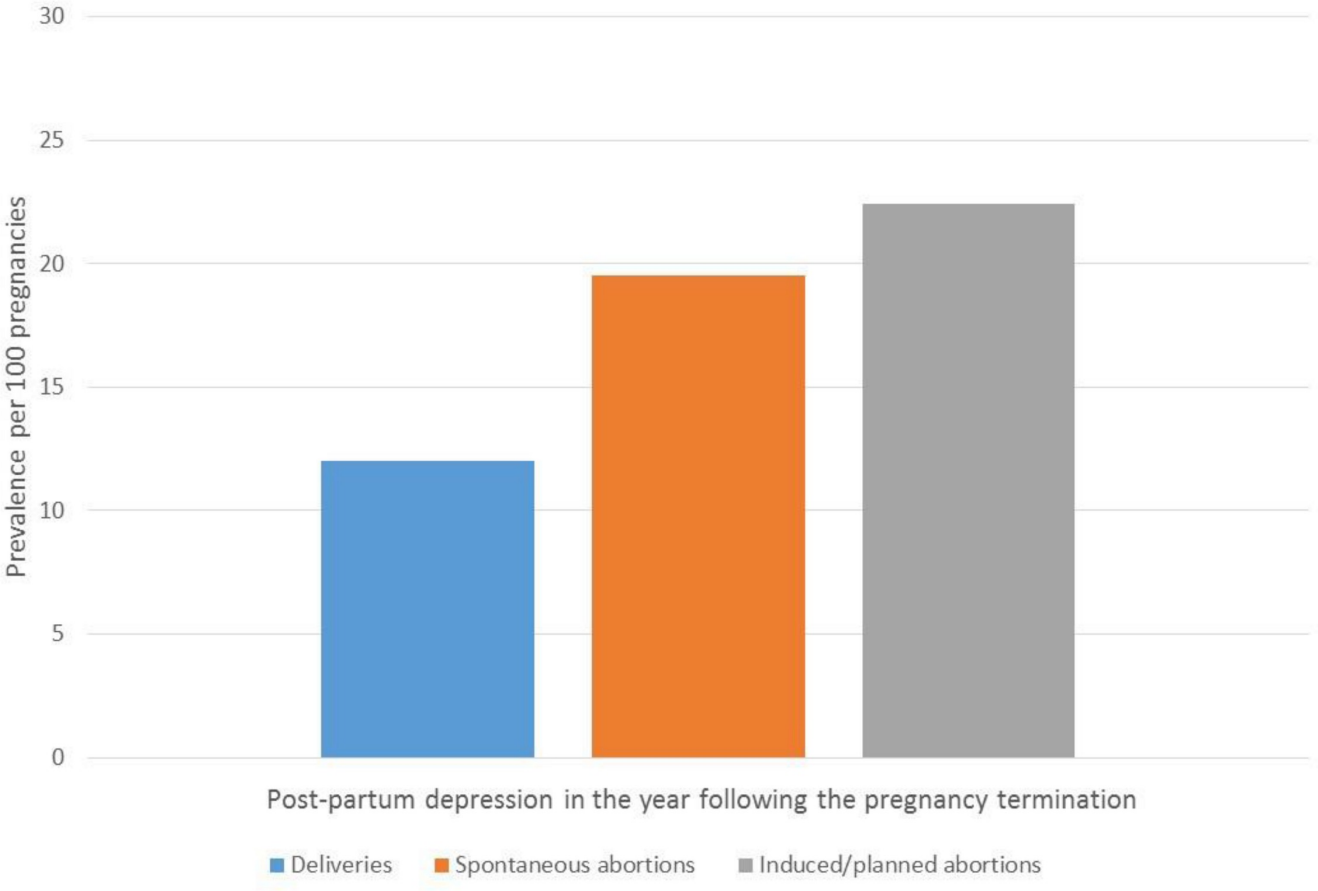
FPC–Prevalences of post-partum depression according to pregnancy outcome.

With regards to four studied maternal comorbidities in the year before or during pregnancy, depression was the most prevalent (19.2%-29.9%), followed by asthma (11.6%-14.9%), diabetes (1.1%-7.7%), and hypertension (0.7%-9.0%) regardless of pregnancy outcome (**Table 1**). Post-partum depression was the highest among pregnancies resulting in stillbirths (38.8%) (**Table 1**). Although it remained high, the prevalence of post-partum depression was the lowest among pregnancies ending with a liveborn infant (12.0%) (**Table 1**). All maternal comorbidities studied, including post-partum depression in the year following the end of pregnancy, varied according to pregnancy outcomes (delivery with livebirths or stillbirths, spontaneous or induced/planned abortion). A higher prevalence of asthma, diabetes, or hypertension was observed in pregnancies resulting in deliveries compared to spontaneous or induced/planned abortion (p = 0.03) (**Table 1**, **Fig 4**). However, maternal depression during and in the 1-year post-partum period was higher among those with spontaneous or planned abortions or stillbirths (**Table 1**, **Figs 4 and 5**) (p = 0.02 for gestational exposure and post-partum). It remains however that maternal depression during pregnancy among those who delivered a liveborn infant was high at 19.2 (**Table 1**, **Fig 4**); post-partum depression in this sub-group was 12.0% (**Table 1**, **Fig 5**).

### Medication use before, during, and after pregnancy, and by trimester in the FPC

**Fig 6:**
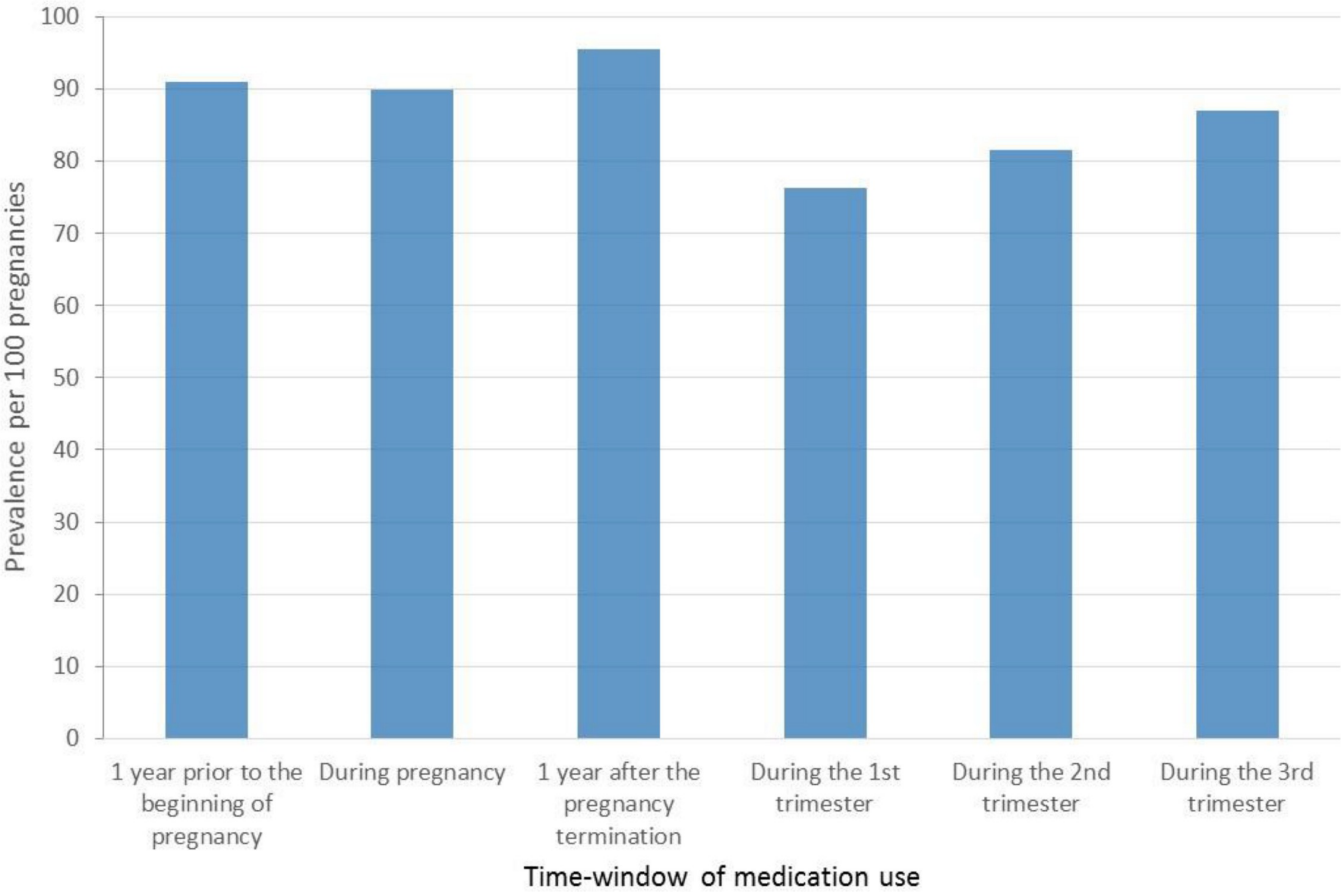
FPC–Prevalence of medication use, stratified by time-window of interest and trimester.

In the FPC, 91.1% of women took medications in the year before their pregnancy, which is one of the highest prevalence of exposure worldwide [3–5, 14] (**Table 1**, **Fig 6**); women filled on average 10.8 prescriptions (SD 9.6) during this time-window (**Table 1**). Once the pregnancy was diagnosed, the prevalence of exposure varied between trimesters, 76.4% in the first trimester, 81.1% in the second, and 88.6% in the third (**Table 1**, **Fig 6**); the mean number of filled prescriptions also decreased (3–4 on average) during the gestational period (**Table 1**). Prevalence of exposure to medications during pregnancy also varied according to pregnancy outcome, with the highest prevalence seen in livebirths (97.3%) and stillbirths (96.5%) (p = 0.04) (**Table 1**). Although it is expected that women with spontaneous or induced/planned abortions are less likely to receive medications due to their shorter gestational age, first-trimester prevalence remained lower in these two sub-groups (74.2% for spontaneous abortion and 64.1% for induced/planned abortions vs. 79.6% for livebirths and 84.1% for stillbirths) (p = 0.04) (**Table 1**). In the year after the end of pregnancy, the prevalence of medication use increased to 95.6% (**Table 1** and **Fig 6**).

**Fig 7:**
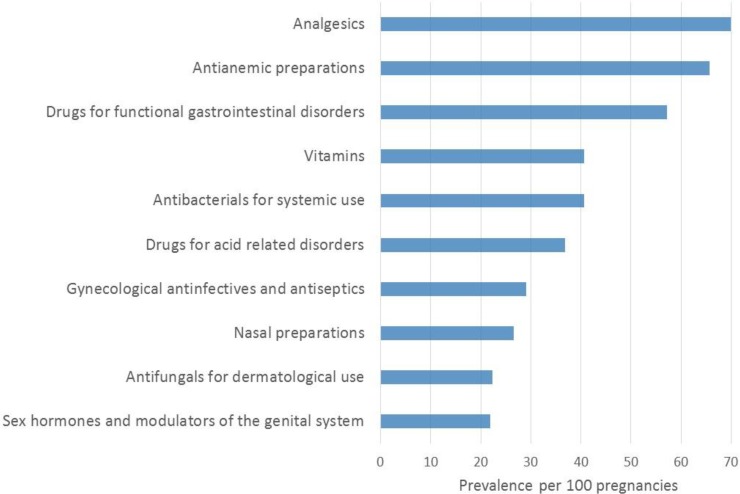
FPC–Top 10 most used medication classes during pregnancy.

**Fig 8:**
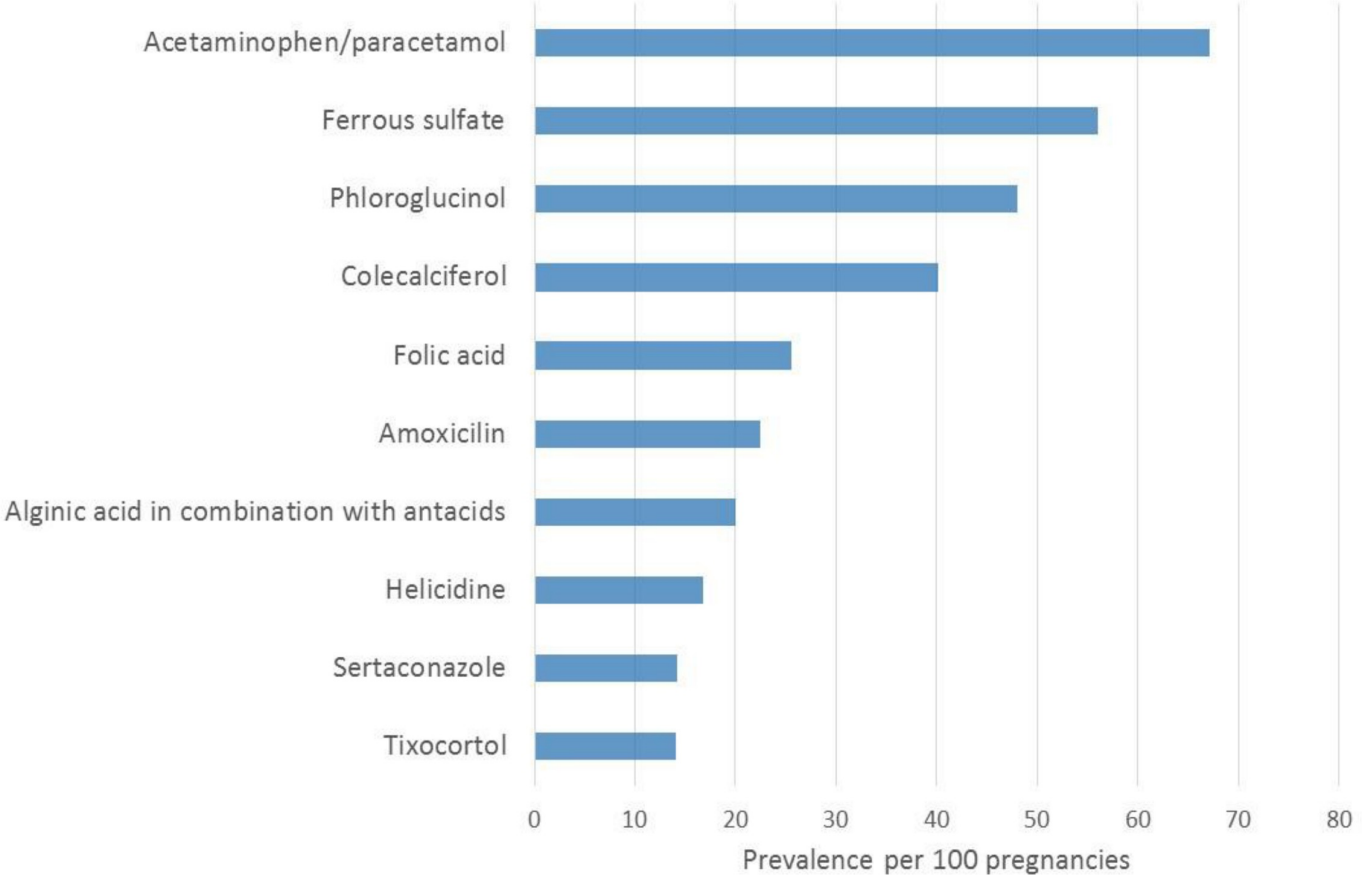
FPC–Top 10 most used medication types during pregnancy.

The top 50 medication classes used, stratified by pregnancy outcomes, and time-window of interest (1 year before, during, and 1 year after pregnancy as well as by trimester) are presented in the **Tables A-F in S1 File**; the top 50 medication types most used according to pregnancy outcomes and time-window of interest are also presented in the **Tables G-L in S1 File**. During pregnancy, analgesics were the most used (70%) (**Fig 7**) followed by antianemia preparations (65.7%), medication for gastrointestinal problems (57.5%), and prescribed and reimbursed vitamins (40.6%). More specifically, acetaminophen was the most used (67.1%) (**Fig 8**), followed by iron (ferrous sulphate) (56.0%), phloroglucinol (gastrointestinal pain) (48.0%), colecalciferol (vitamin D) (40.1%), folic acid (26%), amoxicillin (antibiotics) (22.5%), antacids/acid reflux medications (20%), helicidine (antitussive) (17%), sertaconazole (antifungal) (14%), tixocortol (corticosteroid) (13.9%), metoclopramide (anti-nausea) (13.8%), econazole (antifungal) (13%), domperidone (anti-nausea) (13%), and omeprazole (proton pump inhibitor, antacid) (12%).

## Discussion

To our knowledge, the FPC is the first representative sample cohort of French pregnant women. The FPC includes data on 36,065 pregnancies; 27,253 (75.6%) deliveries including 201 stillbirths (0.7%), which is comparable to the most recent National French perinatal survey (EPOPé) [20]. The FPC also includes data on 2,094 (5.8%) spontaneous abortions, and 6,718 (18.6%) induced/planned abortions, which makes it the first French cohort with longitudinal data on pregnancy terminations. The FPC highlights the fact that 89.9% of women take medications during pregnancy in France, with analgesics, folic acid, antibiotics, and vitamins being the most frequently used. The prevalence of medication use once the pregnancy is diagnosed slightly decreases but increases above the pre-pregnancy level in the year following the end of pregnancy, regardless of pregnancy outcome. Maternal depression was the most frequent maternal comorbidity (20%); post-partum depression was present in 12.0%-38.8% of women depending on whether they had a spontaneous or induced/planned abortion, stillborn or liveborn.

FPC’s maternal characteristics were comparable to the overall French population of pregnant women. Indeed, using the 2016 National French perinatal survey [20], we were able to determine that our prevalence of singleton livebirths was comparable to the national estimate (98% in FPC vs. 98.2% in the national survey); stillbirths occurred in 0.7% of pregnancies in FPC versus 0.6% of pregnancies in the survey; the majority of pregnant women was between 20–35 years of age (76.2% in FPC, 76.6% in the survey, and 77.5% in the overall PMSI database); prematurity was 6.5% in FPC vs. 6.0% in the national survey; 26% of pregnant women included in FPC were taking folic acid compared to 23% in the national survey; in the FPC, 20% also took folic acid before pregnancy; maternal hypertension prevalence was 4.2% in FPC compared to 4.8% in the national survey; and maternal diabetes was 6.1% in FPC vs. 7.2% in the survey.

In the FPC, 18.6% of pregnant women had an induced/planned abortion. This is lower than population-based estimates reported elsewhere: 35.9% in Quebec, Canada (QPC) [3], and 23.3 per 100 pregnancies in the US [21]. This could partly be explained by cultural differences, believes, or maternal preferences. Six percent (5.8%) of pregnant women in the FPC had a clinically diagnosed spontaneous abortion, which is somewhat lower than elsewhere (10%–15%) [22, 23]. Categorization of spontaneous or induced/planned abortions within the FPC is made with specific procedure codes, which limits any potential outcome misclassification (over-estimation of spontaneous abortions and underestimation of induced/planned abortions) that could result from patient or physician reported assessment of outcome in other settings. Nevertheless, differences between prevalence estimates of spontaneous or induced/planned abortions in the FPC and estimates from other cohorts could partly be explained by health care access/systems or by differing lifestyles or environmental exposures including medications.

Within the FPC, prematurity was estimated at 6.5%, which is similar to what has been reported in the United Kingdom (6.5%) [24], but lower than in Belgium (8.4%) [24], Quebec (7.1%) [3], or in the US (10.8%) [25]. FPC’s prevalence of multiplicity is comparable to what has been reported in Canada (2.0% in FPC vs. 2.9% in Canada) [26].

The FPC showed that 89.9% of pregnant women had an on-going medication prescription during gestation. Although there is inter-country variation in the prevalence of medication exposure during gestation (86% The Netherlands, 96% Germany, 74%–100% in other French regional cohorts, 68%–100% USA, 46%–100% Finland, 44% Denmark, 56.6% in Quebec) [3–5, 14, 27], partly explained by cultural differences, lifestyles, drug reimbursement plans, definitions of drug exposure within studies, or maternal age or other maternal characteristics, it remains that the FPC highlights the extent of medication exposure during pregnancy in France. Nevertheless, FPC’s prevalence of gestational exposure to medications is comparable to other French cohorts or pregnant women. Indeed, the overall prevalence of prescribed medication during pregnancy found in our cohort is similar to what has been reported by Lacroix *et al*. [6, 7] and Damase-Michel *et al*. [28] in the EFEMERIS cohort of the region of Haute-Garonne with some disparities between drugs. For example, whereas the rates of anti-nausea drugs were similar between both studies (13.8% vs. 12.9% for metoclopramide and 13% vs. 10.2% for domperidone in FPC and EFEMERIS, respectively), pregnant women in the FPC were using more folic acid (26.0% vs. 11.4% for EFEMERIS), and amoxicillin (first-line treatment for infections during pregnancy, antibiotics) (22.5% vs. 6.1% in EFEMERIS). This could be explained by the fact that FPC’s medication exposure prevalence was calculated on the overall duration of pregnancy, including fillings with duration overlapping the first day of pregnancy whereas only fillings during pregnancy are considered in EFEMERIS. Using the overall duration of gestation to calculate medication exposure prevalence, the FPC is comparable to the Quebec Pregnancy Cohort, which reports 26.5% prevalence of use for antibiotics [29]. The FPC has a higher prevalence of antidepressant use compared to elsewhere in the world (8.7% in FPC vs. 4.9% in the QPC) [3, 30], and other French studies [31, 32]. The variation between study estimates could partly be explained by, as mentioned above, the differences in definitions of measurement of medication exposure during pregnancy between the different databases.

Finally, 12.0–38.8% of pregnant women in the FPC had post-partum depression disorders diagnosed in the year following the end of pregnancy. Post-partum depression was highest for those who delivered a stillborn infant (38.8%), and lowest in those who delivered a liveborn (12.0%). This is comparable to studies which reported rates of postnatal depression disorders of 10.4% at 6-months postpartum in deliveries with liverborns [33]. The FPC gives the opportunity to assess post-partum depression among all pregnant women regardless of pregnancy outcome, which is important given the varying prevalence among groups.

Although there has been an increase in the assembly of cohorts of pregnant women over the past years, the FPC offers an interesting range of variables, and is one of a few that focuses on maternal health in addition of giving valid gestational age, which is essential in perinatal pharmacoepidemiologic studies. The FPC is a representative sample of French pregnant women followed within the universal health care system in France, built with data from the French administrative database (EGB), which includes information from routine billing data and hospital database (PMSI), and includes physician-based prospective diagnoses and procedure codes, data on prescription fillings including date of filling, duration of prescription and dosage, and has data on maternal comorbidities during pregnancy, and pregnancy outcomes. Given the prospective nature of the data collected on prescription fillings, information on medication use do not suffer from recall bias, and appropriate medication filling algorithms can limit bias resulting from drug non-compliance. Because of the administrative nature of the databases used, data on smoking, alcohol and illicit drug use as well as caffeine intake, and maternal weight and weight gain during pregnancy are missing. Although this is a limitation, using appropriate study designs and medication filling algorithms can circumvent it. This study suffers from some limitations inherent in the use of reimbursement databases. As drug dispensation was used as a proxy for drug exposure, we never know whether patients are actually taking their medications. However, De Jonge *et al*. [34] found more than 90% compliance during pregnancy, in particular for chronic treatment. Furthermore, Zhao *et al*. [35] have found that prescription fillings during pregnancy in administrative database have good positive predictive values [35]. In addition, register data on maternal health outcomes is a ‘proxy’ measure, and probably underestimating of the real world situation. Finally, the exact admission, discharge, and medical procedure dates are routinely recorded in the PMSI database for 97.0% of all births and 91.7% of all inpatient induced abortion [36]. In our study the proposed algorithm to identify pregnancies was based on ICD-10 diagnostic codes and medical procedure codes that may be subject to coding errors. However, as the PMSI database is used for planning and funding purposes and is subject to coding quality control, coding errors should therefore be limited [10–12, 37]. In addition, our algorithm is similar to Blotière *et al*. [36], which increases validity.

Finally, we have shown that baseline characteristics from the French Pregnancy Cohort (FPC) were comparable, for the most part, to similar statistics from the overall population of pregnant women in France as well as other pregnancy cohorts or populations published elsewhere. Baseline results presented here mostly highlighted the fact that a high prevalence of pregnant women take prescribed medications during gestation and that more research needs to be done in this special population to fully assess and quantify the risks and benefits of medication exposure for the mother and child. The FPC is a pregnancy cohort that has the potential to fill this knowledge gap.

## Conclusion

In conclusion, the FPC has the advantage of including a representative sample of French pregnant women, and study medications only available in France in addition to others available worldwide. The large number of pregnancies in the cohort provides the power needed to measure maternal pregnancy outcomes. The FPC provides information to measure potential confounding variables, especially valid gestational age, which ensures accurate timing of drug exposure.

We believe that the FPC, built with data from a representative sample of the French population (EGB), is a good tool to study medication exposure during pregnancy and its effect on the mother and children. Given the observational nature of studies on the impact of medication use during pregnancy, replication of findings is key to causality assessment. Hence, the FPC has its place in the epidemiological landscape.

## Supporting information

S1 File Including Appendices A-B and Tables A-L.
(DOC)
