# Supplementary material for: The French Pregnancy Cohort: Medication use during pregnancy in the French population
Source: PLoS One. 2019 Jul 17;14(7):e0219095. doi: 10.1371/journal.pone.0219095 (PMC6636733; doi:10.1371/journal.pone.0219095)
Supplement: S1 File — Including Appendices A-B and Tables A-L. (DOC) [file pone.0219095.s001.doc]

**Support Information**

This appendix has been provided by the authors to give readers additional information about their work.

**Supplementary to**: The French Pregnancy Cohort: medication use during pregnancy in the French population. Bérard A, Abbas F, Kassai B, Vial T, Nguyen KA, Sheehy O, Schott AM.

**Contents:**

**Appendix A.** Algorithm for identification of stays for delivery or miscarriage/abortion.

**Appendix B.** CCMA acts specific to selection of deliveries (DEL).

**Table A.** Top 50 medication classes use in the year prior to pregnancy (yes vs. no): stratified by pregnancy outcomes.

**Table B.** Top 50 medication classes use during pregnancy (yes vs. no): stratified by pregnancy outcomes

**Table C.** Top 50 medication classes use in the year following the pregnancy (yes vs. no): stratified by pregnancy outcomes

**Table D.** Top 50 medication classes use during the first trimester of pregnancy (yes vs. no): stratified by pregnancy outcomes

**Table E.** Top 50 medication classes use during the second trimester of pregnancy (yes vs. no): stratified by pregnancy outcomes

**Table F.** Top 50 medication classes use during the third trimester of pregnancy (yes vs. no): stratified by pregnancy outcomes

**Table G.** Top 50 medication use in the year prior to pregnancy (yes vs. no): stratified by pregnancy outcomes

**Table H.** Top 50 medication use during pregnancy (yes vs. no): stratified by pregnancy outcomes

**Table I.** Top 50 medication use in the year following the pregnancy (yes vs. no): stratified by pregnancy outcomes

**Table J.** Top 50 medication use during the first trimester of pregnancy (yes vs. no): stratified by pregnancy outcomes

**Table K.** Top 50 medication use during the second trimester of pregnancy (yes vs. no): stratified by pregnancy outcomes

**Table L.** Top 50 medication use during the third trimester of pregnancy (yes vs. no): stratified by pregnancy outcomes

**Appendix A. Algorithm for identification of stays for delivery or miscarriage/abortion**

The statistical unit is pregnancy, a woman can be in one or two states during the year studied.

**Selection of deliveries (DEL):** deliveries are most often hospitalized. From the Information Systems Medicalization Program (ISMP, from *Programme de médicalisation des systèmes d'information*), selection of beneficiaries

hospitalized during a stay with a main diagnosis or a related diagnosis or an associated diagnosis, from International Classification of Diseases Version 10 (ICD10), specific to delivery: See list of DEL codes appendix 1.

**or**

hospitalized during a stay with at least one act CCAM (Common Classification of Medical Acts) specific to childbirth among the list of acts "DEL", See list of appendix codes 2.

**Selection of abortions and miscarriages (ABORT/Miscarriage):** voluntary pregnancy interruptions, medical pregnancy interruptions, or miscarriages are not always hospitalized except complications. From the ISMP, selection of beneficiaries

hospitalized during a stay with a main diagnosis or a related diagnosis or associated diagnosis, from ICD10, specific to abortion or miscarriage: cf. list of "DEL/Miscarriages" codes appendix 1.

**or**

hospitalized during a stay with at least one act CCAM (Common Classification of Medical Acts) specific to abortion or miscarriage among the list of acts "ABORT/Miscarriage", see list of appendix codes 2.

In the case where a beneficiary is in more than two states during the same year, the beneficiary's data are put on a line in order to arrange the different states in time and to distinguish them. If for the same ISMP stay two states "ABORT/Miscarriage" and "DEL" are identified then the stay is classified in the state "ABORT/Miscarriage ".

**DEL-specific ICD-10 diagnostics:**

- O151: Eclampsia in labour
  - - O30: Multiple gestation
    - O30.0: Twin pregnancy
    - O30.1: Triplet pregnancy
    - O30.2: Quadruplet pregnancy
    - O30.8: Other multiple gestation
    - O30.9: Multiple gestation, unspecified
    - O32: Maternal care for known or suspected malpresentation of fetus
    - O32.0: Maternal care for unstable lie
    - O32.1: Maternal care for breech presentation
    - O32.2: Maternal care for transverse and oblique lie
    - O32.3: Maternal care for face, brow and chin presentation
    - O32.4: Maternal care for high head at term
    - O32.5: Maternal care for multiple gestation with malpresentation of one fetus or more
    - O32.6: Maternal care for compound presentation
    - O32.8: Maternal care for other malpresentation of fetus
    - O32.9: Maternal care for malpresentation of fetus, unspecified
    - O33.5: Maternal care for disproportion due to unusually large fetus
    - O33.6: Maternal care for disproportion due to hydrocephalic fetus
    - O33.7: Maternal care for disproportion due to other fetal deformities
    - O40: Polyhydramnios
    - O41: Other disorders of amniotic fluid and membranes
    - O41.1: Infection of amniotic sac and membranes
    - O41.8: Other specified disorders of amniotic fluid and membranes
    - O41.9: Disorder of amniotic fluid and membranes, unspecified
    - O42: Premature rupture of membranes
    - O42.0: Premature rupture of membranes, onset of labour within 24 hours
    - O42.1: Premature rupture of membranes, onset of labour after 24 hours
    - O44: Placenta praevia
    - O44.0: Placenta praevia specified as without haemorrhage
    - O44.1: Placenta praevia with haemorrhage
    - O45: Premature separation of placenta [abruptio placentae]
    - O45.0: Premature separation of placenta with coagulation defect
    - O45.8: Other premature separation of placenta
    - O45.9: Premature separation of placenta, unspecified
    - O46: Antepartum haemorrhage, not elsewhere classified
    - O46.0: Antepartum haemorrhage with coagulation defect
    - O46.8: Other antepartum haemorrhage
    - O46.9: Antepartum haemorrhage, unspecified
    - O47.1: False labour at or after 37 completed weeks of gestation
    - O48: Prolonged pregnancy
- O60: Preterm labour and delivery
  - - O60.0: Preterm labour without delivery
    - O60.1: Preterm spontaneous labour with preterm delivery
    - O60.2: Preterm spontaneous labour with term delivery
    - O60.3: Preterm delivery without spontaneous labour
- O61.0: Failed medical induction of labour
- O61.1: Failed instrumental induction of labour
- O61.8: Other failed induction of labour
- O61.9: Failed induction of labour, unspecified
- O62.0: Primary inadequate contractions
- O62.1: Secondary uterine inertia
- O62.2: Other uterine inertia
- O62.3: Precipitate labour
- O62.4: Hypertonic, incoordinate, and prolonged uterine contractions
- O62.8: Other abnormalities of forces of labour
- O62.9: Abnormality of forces of labour, unspecified
- O63.0: Prolonged first stage (of labour)
- O63.1: Prolonged second stage (of labour)
- O63.2: Delayed delivery of second twin, triplet, etc.
- O63.9: Long labour, unspecified
- O64.0: Obstructed labour due to incomplete rotation of fetal head
- O64.1: Obstructed labour due to breech presentation
- O64.2: Obstructed labour due to face presentation
- O64.3: Obstructed labour due to brow presentation
- O64.4: Obstructed labour due to shoulder presentation
- O64.5: Obstructed labour due to compound presentation
- O64.8: Obstructed labour due to other malposition and malpresentation
- O64.9: Obstructed labour due to malposition and malpresentation, unspecified
- O65.0: Obstructed labour due to deformed pelvis
- O65.1: Obstructed labour due to generally contracted pelvis
- O65.2: Obstructed labour due to pelvic inlet contraction
- O65.3: Obstructed labour due to pelvic outlet and mid-cavity contraction
- O65.4: Obstructed labour due to fetopelvic disproportion, unspecified
- O65.5: Obstructed labour due to abnormality of maternal pelvic organs
- O65.8: Obstructed labour due to other maternal pelvic abnormalities
- O65.9: Obstructed labour due to maternal pelvic abnormality, unspecified
- O66.0: Obstructed labour due to shoulder dystocia
- O66.1: Obstructed labour due to locked twins
- O66.2: Obstructed labour due to unusually large fetus
- O66.3: Obstructed labour due to other abnormalities of fetus
- O66.4: Failed trial of labour, unspecified
- O66.5: Failed application of vacuum extractor and forceps, unspecified
- O66.8: Other specified obstructed labour
- O66.9: Obstructed labour, unspecified
- O67.0: Intrapartum haemorrhage with coagulation defect
- O67.8: Other intrapartum haemorrhage
- O67.9: Intrapartum haemorrhage, unspecified
- O68.0: Labour and delivery complicated by fetal heart rate anomaly
- O68.1: Labour and delivery complicated by meconium in amniotic fluid
- O68.2: Labour and delivery complicated by fetal heart rate anomaly with meconium in amniotic fluid
- O68.3: Labour and delivery complicated by biochemical evidence of fetal stress
- O68.8: Labour and delivery complicated by other evidence of fetal stress
- O68.9: Labour and delivery complicated by fetal stress, unspecified
- O69.0: Labour and delivery complicated by prolapse of cord
- O69.1: Labour and delivery complicated by cord around neck, with compression
- O69.2: Labour and delivery complicated by other cord entanglement, with compression
- O69.3: Labour and delivery complicated by short cord
- O69.4: Labour and delivery complicated by vasa praevia
- O69.5: Labour and delivery complicated by vascular lesion of cord
- O69.8: Labour and delivery complicated by other cord complications
- O69.9: Labour and delivery complicated by cord complication, unspecified
- O70.0: First degree perineal laceration during delivery
- O70.1: Second degree perineal laceration during delivery
- O70.2: Third degree perineal laceration during delivery
- O70.3: Fourth degree perineal laceration during delivery
- O70.9: Perineal laceration during delivery, unspecified
- O71.0: Rupture of uterus before onset of labour
- O71.1: Rupture of uterus during labour
- O71.2: Postpartum inversion of uterus
- O71.3: Obstetric laceration of cervix
- O71.4: Obstetric high vaginal laceration
- O71.5: Other obstetric injury to pelvic organs
- O71.6: Obstetric damage to pelvic joints and ligaments
- O71.7: Obstetric haematoma of pelvis
- O71.8: Other specified obstetric trauma
- O71.9: Obstetric trauma, unspecified
- O72.0: Third-stage haemorrhage
- O72.1: Other immediate postpartum haemorrhage
- O72.2: Delayed and secondary postpartum haemorrhage
- O72.3: Postpartum coagulation defects
- O73.0: Retained placenta without haemorrhage
- O73.1: Retained portions of placenta and membranes, without haemorrhage
- O74.0: Aspiration pneumonitis due to anaesthesia during labour and delivery
- O74.1: Other pulmonary complications of anaesthesia during labour and delivery
- O74.2: Cardiac complications of anaesthesia during labour and delivery
- O74.3: Central nervous system complications of anaesthesia during labour and delivery
- O74.4: Toxic reaction to local anaesthesia during labour and delivery
- O74.5: Spinal and epidural anaesthesia-induced headache during labour and delivery
- O74.6: Other complications of spinal and epidural anaesthesia during labour and delivery
- O74.7: Failed or difficult intubation during labour and delivery
- O74.8: Other complications of anaesthesia during labour and delivery
- O74.9: Complication of anaesthesia during labour and delivery, unspecified
- O75.0: Maternal distress during labour and delivery
- O75.1: Shock during or following labour and delivery
- O75.2: Pyrexia during labour, not elsewhere classified
- O75.3: Other infection during labour
- O75.4: Other complications of obstetric surgery and procedures
- O75.5: Delayed delivery after artificial rupture of membranes
- O75.6: Delayed delivery after spontaneous or unspecified rupture of membranes
- O75.7: Vaginal delivery following previous caesarean section
- O75.8: Other specified complications of labour and delivery
- O75.9: Complication of labour and delivery, unspecified
- O80: Single spontaneous delivery
- O80.0: Spontaneous vertex delivery
- O80.1: Spontaneous breech delivery
- O80.8: Other single spontaneous delivery
- O80.9: Single spontaneous delivery, unspecified
- O81.0: Low forceps delivery
- O81.1: Mid-cavity forceps delivery
- O81.2: Mid-cavity forceps with rotation
- O81.3: Other and unspecified forceps delivery
- O81.4: Vacuum extractor delivery
- O81.5: Delivery by combination of forceps and vacuum extractor
- O82.0: Delivery by elective caesarean section
- O82.1: Delivery by emergency caesarean section
- O82.2: Delivery by caesarean hysterectomy
- O82.8: Other single delivery by caesarean section
- O82.9: Delivery by caesarean section, unspecified
- O83.0: Breech extraction
- O83.1: Other assisted breech delivery
- O83.2: Other manipulation-assisted delivery
- O83.3: Delivery of viable fetus in abdominal pregnancy
- O83.4: Destructive operation for delivery
- O83.8: Other specified assisted single delivery
- O83.9: Assisted single delivery, unspecified
- O84.0: Multiple delivery, all spontaneous
- O84.1: Multiple delivery, all by forceps and vacuum extractor
- O84.2: Multiple delivery, all by caesarean section
- O84.8: Other multiple delivery
- O84.9: Multiple delivery, unspecified
- Z370 Single live birth
- Z371 Single stillbirth
- Z372 Twins, both liveborn
- Z373 Twins, one liveborn and one stillborn
- Z374 Twins, both stillborn
- Z375 Other multiple births, all liveborn
- Z376 Other multiple births, some liveborn
- Z377 Other multiple births, all stillborn
- Z379 Outcome of delivery, unspecified
- Z380 Singleton, born in hospital
- Z381 Singleton, born outside hospital
- Z382 Singleton, unspecified as to place of birth
- Z383 Twin, born in hospital
- Z384 Twin, born outside hospital
- Z385 Twin, unspecified as to place of birth
- Z386 Other multiple, born in hospital
- Z387 Other multiple, born outside hospital
- Z388 Other multiple, unspecified as to place of birth
- Z390 Care and examination immediately after delivery
- Z391 Care and examination of lactating mother
- Z392 Routine postpartum follow-up

**ABORT/Miscarriage-specific ICD-10 diagnostics:**

- O00.0: Abdominal pregnancy
- O00.1: Tubal pregnancy
- O00.2: Ovarian pregnancy
- O00.8: Other ectopic pregnancy
- O00.9: Ectopic pregnancy, unspecified
- O01.0: Classical hydatidiform mole
- O01.1: Incomplete and partial hydatidiform mole
- O01.9: Hydatidiform mole, unspecified
- O02.0: Blighted ovum and nonhydatidiform mole
- O02.1: Missed abortion
- O02.8: Other specified abnormal products of conception
- O02.9: Abnormal product of conception, unspecified
- O03.0: Spontaneous abortion, Incomplete, complicated by genital tract and pelvic infection
- O03.1: Spontaneous abortion, Incomplete, complicated by delayed or excessive haemorrhage
- O03.2: Spontaneous abortion, Incomplete, complicated by embolism
- O03.3: Spontaneous abortion, Incomplete, with other and unspecified complications
- O03.4: Spontaneous abortion, Incomplete, without complication
- O03.5: Spontaneous abortion, Complete or unspecified, complicated by genital tract and pelvic infection
- O03.6: Spontaneous abortion, Complete or unspecified, complicated by delayed or excessive haemorrhage
- O03.7: Spontaneous abortion, Complete or unspecified, complicated by embolism
- O03.8: Spontaneous abortion, Complete or unspecified, with other and unspecified complications
- O03.9: Spontaneous abortion, Complete or unspecified, without complication
- O04.0: Medical abortion, Incomplete, complicated by genital tract and pelvic infection
- O04.1: Medical abortion, Incomplete, complicated by delayed or excessive haemorrhage
- O04.2: Medical abortion, Incomplete, complicated by embolism
- O04.3: Medical abortion, Incomplete, with other and unspecified complications
- O04.4: Medical abortion, Incomplete, without complication
- O04.5: Medical abortion, Complete or unspecified, complicated by genital tract and pelvic infection
- O04.6: Medical abortion, Complete or unspecified, complicated by delayed or excessive haemorrhage
- O04.7:Medical abortion, Complete or unspecified, complicated by embolism
- O04.8: Medical abortion, Complete or unspecified, with other and unspecified complications
- O04.9: Medical abortion, Complete or unspecified, without complication
- O05.0: Other abortion, Incomplete, complicated by genital tract and pelvic infection
- O05.1: Other abortion, Incomplete, complicated by delayed or excessive haemorrhage
- O05.2: Other abortion, Incomplete, complicated by embolism
- O05.3:Other abortion, Incomplete, with other and unspecified complications
- O05.4: Other abortion, Incomplete, without complication
- O05.5:Other abortion, Complete or unspecified, complicated by genital tract and pelvic infection
- O05.6: Other abortion, Complete or unspecified, complicated by delayed or excessive haemorrhage
- O05.7: Other abortion, Complete or unspecified, complicated by embolism
- O05.8: Other abortion, Complete or unspecified, with other and unspecified complications
- O05.9: Other abortion, Complete or unspecified, without complication
- O06.0: Unspecified abortion, Incomplete, complicated by genital tract and pelvic infection
- O06.1: Unspecified abortion, Incomplete, complicated by delayed or excessive haemorrhage
- O06.2: Unspecified abortion, Incomplete, complicated by embolism
- O06.3: Unspecified abortion, Incomplete, with other and unspecified complications
- O06.4: Unspecified abortion, Incomplete, without complication
- O06.5: Unspecified abortion, Complete or unspecified, complicated by genital tract and pelvic infection
- O06.6: Unspecified abortion, Complete or unspecified, complicated by delayed or excessive haemorrhage
- O06.7: Unspecified abortion, Complete or unspecified, complicated by embolism
- O06.8: Unspecified abortion, Complete or unspecified, with other and unspecified complications
- O06.9: Unspecified abortion, Complete or unspecified, without complication
- O08.0: Genital tract and pelvic infection following abortion and ectopic and molar pregnancy
- O08.1: Delayed or excessive haemorrhage following abortion and ectopic and molar pregnancy
- O08.2: Embolism following abortion and ectopic and molar pregnancy
- O08.3: Shock following abortion and ectopic and molar pregnancy
- O08.4: Renal failure following abortion and ectopic and molar pregnancy
- O08.5: Metabolic disorders following abortion and ectopic and molar pregnancy
- O08.6: Damage to pelvic organs and tissues following abortion and ectopic and molar pregnancy
- O08.7: Other venous complications following abortion and ectopic and molar pregnancy
- O08.8: Other complications following abortion and ectopic and molar pregnancy
- O08.9: Complication following abortion and ectopic and molar pregnancy, unspecified
- O36.4: Maternal care for intrauterine death

**Appendix B. CCMA acts specific to DEL**

Obstetrical acts during the prenatal period:

- JNBD001 – Cervical cerclage during pregnancy, by transvaginal route
- JNGD002 – Cervical cerclage ablation
- JPJB001 – Drainage of amniotic liquid for hydramnios, by transcutaneous route
- JPLB001 – Amnioinfusion, by transcutaneous route

Notes: Activity 1: ultrasound guidance Activity 2: amnioinfusion

- JQEP001 – Version of the fetus by external obstetric maneuvers during pregnancy, with ultrasound control and monitoring of fetal heart rate

Obstetrical acts during labour and delivery

[Delivery](http://www.ameli.fr/accueil-de-la-ccam/trouver-un-acte/consultation-par-chapitre.php?chap=a%3A5%3A{i%3A0%3Bs%3A7%3A"9.3.3.1"%3Bi%3A1%3Bs%3A7%3A"9.3.3.2"%3Bi%3A2%3Bs%3A7%3A"9.3.4.1"%3Bi%3A3%3Bs%3A7%3A"9.3.4.2"%3Bi%3A4%3Bs%3A7%3A"9.3.4.3"%3B}&del=9.3.3.1" \l "chapitre_9.3.3.1) by natural route

- JQGD001 – Single breech delivery by natural route, in a multiparous woman

Notes: Vermelin method, in a multiparous woman

- JQGD002 – Multiple delivery by natural route, in a primiparous woman
- JQGD003 - Single breech delivery by natural route with small extraction, in a primiparous woman
- JQGD004 – Single breech delivery by natural route, in a primiparous woman

Notes : Vermelin method, in a primiparous woman

- JQGD005 – Single breech delivery by natural route with large extraction, in a multiparous woman
- JQGD007 – Multiple delivery by natural route, in a multiparous woman
- JQGD008 – Single breech delivery by natural route with small extraction, in a multiparous woman
- JQGD010 – Single cephalic delivery by natural route, in a primiparous woman
- JQGD012 – Single cephalic delivery by natural route, in a multiparous woman
- JQGD013 – Single breech delivery by natural route with large extraction, in a primiparous woman

[Ceasarean](http://www.ameli.fr/accueil-de-la-ccam/trouver-un-acte/consultation-par-chapitre.php?chap=a%3A5%3A{i%3A0%3Bs%3A7%3A"9.3.3.1"%3Bi%3A1%3Bs%3A7%3A"9.3.3.2"%3Bi%3A2%3Bs%3A7%3A"9.3.4.1"%3Bi%3A3%3Bs%3A7%3A"9.3.4.2"%3Bi%3A4%3Bs%3A7%3A"9.3.4.3"%3B}&del=9.3.3.2" \l "chapitre_9.3.3.2) delivery

- JQGA002 – Scheduled caesarean delivery, by laparotomy
- JQGA003 – Ceasarean delivery during labour, by laparotomy
- JQGA004 – Emergency ceasarean delivery outside of labour, by laparotomy
- JQGA005 – Ceasarean delivery, by a vaginal approach

Treatment of immediate complications of childbirth

Immediate suture of maternal obstetric lesions

- JMCA001 – Immediate suture of obstetric tearing of the perineum with lesion of the rectum [complicated complete perineum]
- JMCA002 – Immediate suture of obstetric tearing of the vagina, vulva and/or perineum [simple perineum]
- JMCA003 – Immediate suture of obstetric tearing of the perineum with lesion of the external anal sphincter [complete perineum]
- JMCA004 – Immediate suture of obstetric tearing of the perineum with bladder or urethral lesion
- JNCA001 – Immediate suture of obstetrical cervical tearing
- JNCA002 – Suture of the body of the uterus [Hysterography] for obstetric rupture, by laparotomy

Acts of general or locoregional anesthesia, complementary to a diagnostic or therapeutic act; spinal anesthesia for childbirth

- AFLB010 – Spinal anaesthesia during vaginal delivery

Notes : Billing : Cannot be billed for an anaesthesia for a caesarean delivery under the subparagraph 09.03.03.02; cannot be billed with a complementary locoregional anaesthesia level 1, 2, 3, 4, 5, or 6

Complimentary acts related to pregnancy and delivery

- JMPA006 – Episiotomy

Notes: Episiotomy procedure and repair

- JNMD002 – Examination of the uterine cavity after natural delivery
- JNQD001 – Electronic monitoring of contractions of the pregnant uterus and/or fetal heart rate, by uterine route

Notes: With or without transcutaneous monitoring

- JPGD001 – Manual extraction of the complete placenta

Notes: Artificial delivery with uterine examination

- JQED001 – Version of the fetus by internal obstetric maneuvers

Notes: Indication: assisted birth of a fetus in a multiple pregnancy following a vaginal birth of the first infant. This can allow the vaginal delivery of a stillborn fetus. Environment: nearby and available operating room in order to be able to perform a caesarean in the best timeframe

- JQED002 - Reduction of severe shoulder dystocia

Notes: Jacquemier manœuvre

- JQGD006 - Instrumental extraction at the pelvic outlet on cephalic presentation

Notes: Application of forceps, spatulas, obstetric cups to the pelvic outlet

- JQGD009 – Instrumental extraction at the pelvic midplane on cephalic presentation

Notes: Application of forceps, spatulas, obstetric cups to the pelvic midplane. Environment: nearby and available operating room in order to be able to perform a caesarean in the best timeframe

- JQGD011 – Instrumental extraction on head last, during a breech delivery
- JQHB001 – Fetal blood draw on the presentation
- JQQP900 – Transcutaneous oximetry of the fetus on the presentation [fetal pulse oximetry]
- YYYY069 – Supplement for multiple delivery by caesarean
- **CCMA specific to ABORT/miscarriage:**

Acts related to a pregnancy ending by an abortion

- - JJFA001 – Partial or total salpingectomy for ectopic pregnancy, by laparotomy
  - JJFC001 – Partial or total salpingectomy for ectopic pregnancy, by laparoscopy
  - JJJA002 – Tubal expression for tuboabdominal evacuation of ectopic pregnancy, by laparotomy
  - JJJC002 – Tubal expression for tuboabdominal evacuation of ectopic pregnancy, by laparoscopy
  - JJLJ001 – Introvocular injection of pharmacological agent for ectopic pregnancy, by transvaginal route with ultrasound guidance
  - JJPA001 - Salpingotomy with aspiration of ectopic pregnancy, by laparotomy
  - JJPC001 - Salpingotomy with aspiration of ectopic pregnancy, by laparoscopy
  - JNJD001 - Evacuation of a pregnant uterus in the 2nd trimester of pregnancy before the 22nd week of amenorrhea

Notes: Uterine evacuation for retention of dead fetus, for spontaneous miscarriage Therapeutic interruption of pregnancy

- - JNJD002 – Evacuation of a pregnant uterus by aspiration and/or curettage, in the 1st trimester of pregnancy

Notes: Billing: therapeutic interruption of pregnancy; the pricing of voluntary interruptions of pregnancy falls under the decree of July 23, 2004 relating to the fixed rates related to the voluntary termination of pregnancy

- - JNJP001 – Evacuation of a gravid uterus by medication, in the first trimester of pregnancy

Notes: With or without: examination of the uterine cavity. Billing: therapeutic interruption of pregnancy; the pricing of voluntary interruptions of pregnancy falls under the decree of July 23, 2004 relating to the fixed rates related to the voluntary termination of pregnancy

- - JNMD001 – Examination of the uterine cavity after abortion
  - JQGA001 – Abdominal ectopic pregnancy extraction beyond 13 weeks of amenorrhea, by laparotomy

Notes: Excluding abdominal organ excision for abdominal ectopic pregnancy; code only the act of excision.

- - JQGD014 – Selective extraction of fetuses during a multiple pregnancy

Notes: Embryo reduction, with ultrasound guidance

**Table A. Top 50 medication classes use in the year prior to pregnancy (yes vs. no): stratified by pregnancy outcomes**

| **Top 50 medication classes use in the year prior to pregnancy (yes vs. no)** | **All pregnancies 2010 - 2013 (n=32839)** | **Livebirths (n=24877)** | **Stillbirths (n=189)** | **Spontaneous abortions (n=1894)** | **Planned abortions (n=5879)** |
| --- | --- | --- | --- | --- | --- |
| Analgesics | 24374 (74.2 %) | 18364 (73.8 %) | 141 (74.6 %) | 1462 (77.2 %) | 4407 (75.0 %) |
| Antibacterials for systemic use | 19711 (60.0 %) | 14793 (59.5 %) | 114 (60.3 %) | 1171 (61.8 %) | 3633 (61.8 %) |
| Antiinflammatories and antirhumatics | 17074 (52.0 %) | 12837 (51.6 %) | 94 (49.7 %) | 1035 (54.6 %) | 3108 (52.9 %) |
| Sex hormones and modulators of the genital system | 15938 (48.5 %) | 12311 (49.5 %) | 89 (47.1 %) | 912 (48.2 %) | 2626 (44.7 %) |
| Nasal preparations | 13392 (40.8 %) | 10195 (41.0 %) | 65 (34.4 %) | 814 (43.0 %) | 2318 (39.4 %) |
| Drugs for functional gastrointestinal disorders | 12880 (39.2 %) | 9822 (39.5 %) | 78 (41.3 %) | 798 (42.1 %) | 2182 (37.1 %) |
| Antianemic preparations | 10061 (30.6 %) | 8235 (33.1 %) | 53 (28.0 %) | 612 (32.3 %) | 1161 (19.7 %) |
| Drugs for acid related disorders | 9275 (28.2 %) | 7005 (28.2 %) | 49 (25.9 %) | 582 (30.7 %) | 1639 (27.9 %) |
| Cough and cold preparations | 9252 (28.2 %) | 7027 (28.2 %) | 58 (30.7 %) | 541 (28.6 %) | 1626 (27.7 %) |
| Antihistamines for systemic use | 8738 (26.6 %) | 6615 (26.6 %) | 42 (22.2 %) | 532 (28.1 %) | 1549 (26.3 %) |
| Corticosteroids for systemic use | 8108 (24.7 %) | 6127 (24.6 %) | 48 (25.4 %) | 495 (26.1 %) | 1438 (24.5 %) |
| Gynecological antiinfectives and antiseptics | 6559 (20.0 %) | 4853 (19.5 %) | 44 (23.3 %) | 401 (21.2 %) | 1261 (21.4 %) |
| Antifungals for dermatological use | 6433 (19.6 %) | 4854 (19.5 %) | 35 (18.5 %) | 410 (21.6 %) | 1134 (19.3 %) |
| Antiseptics and disinfectants | 5941 (18.1 %) | 4345 (17.5 %) | 39 (20.6 %) | 357 (18.8 %) | 1200 (20.4 %) |
| Psycholeptics | 5545 (16.9 %) | 3910 (15.7 %) | 34 (18.0 %) | 349 (18.4 %) | 1252 (21.3 %) |
| Topical products for joint and muscular pain | 5013 (15.3 %) | 3766 (15.1 %) | 37 (19.6 %) | 338 (17.8 %) | 872 (14.8 %) |
| Stomatological preparations | 4974 (15.1 %) | 3689 (14.8 %) | 35 (18.5 %) | 261 (13.8 %) | 989 (16.8 %) |
| Corticosteroids, dermatological preparations | 4722 (14.4 %) | 3607 (14.5 %) | 29 (15.3 %) | 288 (15.2 %) | 798 (13.6 %) |
| Ophthalmologicals | 4356 (13.3 %) | 3325 (13.4 %) | 28 (14.8 %) | 247 (13.0 %) | 756 (12.9 %) |
| Antidiarrheals, intestinal antiinflammatory/antiinfective agents | 4192 (12.8 %) | 3250 (13.1 %) | 21 (11.1 %) | 235 (12.4 %) | 686 (11.7 %) |
| Vaccines | 3768 (11.5 %) | 2860 (11.5 %) | 24 (12.7 %) | 204 (10.8 %) | 680 (11.6 %) |
| Drugs for obstructive airway diseases | 3729 (11.4 %) | 2794 (11.2 %) | 16 (8.5 %) | 222 (11.7 %) | 697 (11.9 %) |
| Muscle relaxants | 3583 (10.9 %) | 2689 (10.8 %) | 24 (12.7 %) | 235 (12.4 %) | 635 (10.8 %) |
| Vitamins | 3292 (10.0 %) | 2450 (9.8 %) | 19 (10.1 %) | 205 (10.8 %) | 618 (10.5 %) |
| Antibiotics and chemotherapeutics for dermatological use | 3071 (9.4 %) | 2240 (9.0 %) | 18 (9.5 %) | 213 (11.2 %) | 600 (10.2 %) |
| Contact laxatives | 2781 (8.5 %) | 2121 (8.5 %) | 16 (8.5 %) | 146 (7.7 %) | 498 (8.5 %) |
| Antiemetics and antinauseants | 2615 (8.0 %) | 1995 (8.0 %) | 17 (9.0 %) | 150 (7.9 %) | 453 (7.7 %) |
| Emollients and protectives | 2347 (7.1 %) | 1726 (6.9 %) | 11 (5.8 %) | 136 (7.2 %) | 474 (8.1 %) |
| Psychoanaleptics | 2288 (7.0 %) | 1590 (6.4 %) | 12 (6.3 %) | 133 (7.0 %) | 553 (9.4 %) |
| Anti-acne preparations | 2199 (6.7 %) | 1610 (6.5 %) | 14 (7.4 %) | 121 (6.4 %) | 454 (7.7 %) |
| Mineral supplements | 2143 (6.5 %) | 1577 (6.3 %) | 10 (5.3 %) | 142 (7.5 %) | 414 (7.0 %) |
| Contrast media | 2048 (6.2 %) | 1633 (6.6 %) | 17 (9.0 %) | 148 (7.8 %) | 250 (4.3 %) |
| Otologicals | 2018 (6.1 %) | 1514 (6.1 %) | 8 (4.2 %) | 131 (6.9 %) | 365 (6.2 %) |
| Other gynecologicals | 1599 (4.9 %) | 1068 (4.3 %) | 9 (4.8 %) | 97 (5.1 %) | 425 (7.2 %) |
| Antiprotozoals | 1422 (4.3 %) | 1000 (4.0 %) | 6 (3.2 %) | 85 (4.5 %) | 331 (5.6 %) |
| Anesthetics | 1366 (4.2 %) | 1012 (4.1 %) | 6 (3.2 %) | 78 (4.1 %) | 270 (4.6 %) |
| Homeopathic specialties | 1153 (3.5 %) | 853 (3.4 %) | 9 (4.8 %) | 80 (4.2 %) | 211 (3.6 %) |
| Antivirals for systemic use | 1138 (3.5 %) | 831 (3.3 %) | 8 (4.2 %) | 73 (3.9 %) | 226 (3.8 %) |
| Antithrombotic agents | 1071 (3.3 %) | 762 (3.1 %) | 9 (4.8 %) | 75 (4.0 %) | 225 (3.8 %) |
| Other nervous system drugs | 1066 (3.2 %) | 789 (3.2 %) | 4 (2.1 %) | 68 (3.6 %) | 205 (3.5 %) |
| Thyroid preparations | 900 (2.7 %) | 704 (2.8 %) | 8 (4.2 %) | 67 (3.5 %) | 121 (2.1 %) |
| Anthelmintics | 792 (2.4 %) | 551 (2.2 %) | 7 (3.7 %) | 45 (2.4 %) | 189 (3.2 %) |
| Vasoprotective | 732 (2.2 %) | 571 (2.3 %) | 3 (1.6 %) | 32 (1.7 %) | 126 (2.1 %) |
| Antihemorrhagics | 732 (2.2 %) | 527 (2.1 %) | 0 (0%) | 46 (2.4 %) | 159 (2.7 %) |
| Antimycotics for systemic use | 565 (1.7 %) | 436 (1.8 %) | 4 (2.1 %) | 34 (1.8 %) | 91 (1.5 %) |
| Beta blocking agents | 564 (1.7 %) | 415 (1.7 %) | 5 (2.6 %) | 37 (2.0 %) | 107 (1.8 %) |
| Pituitary and hypothalamic hormones and analogues | 543 (1.7 %) | 468 (1.9 %) | 4 (2.1 %) | 48 (2.5 %) | 23 (0.4 %) |
| Antiepileptics | 526 (1.6 %) | 356 (1.4 %) | 3 (1.6 %) | 46 (2.4 %) | 121 (2.1 %) |
| Endocrine therapy | 525 (1.6 %) | 452 (1.8 %) | 6 (3.2 %) | 44 (2.3 %) | 23 (0.4 %) |
| Blood substitutes and perfusion solutions | 498 (1.5 %) | 340 (1.4 %) | 2 (1.1 %) | 34 (1.8 %) | 122 (2.1 %) |

**Note:** The sample size can vary given that some pregnancies did not use medication. A pregnancy can be counted more than once if a woman used more than one medications.

**Table B.** Top 50 medication classes use during pregnancy (yes vs. no): stratified by pregnancy outcomes

| **Top 50 medication classes use during pregnancy (yes vs. no)** | **All pregnancies 2010 - 2013 (n=32408)** | **Livebirths (n=26319)** | **Stillbirths (n=194)** | **Spontaneous abortions (n=1561)** | **Planned abortions (n=4334)** |
| --- | --- | --- | --- | --- | --- |
| Analgesics | 22,691 (70.0 %) | 19525 (74.2 %) | 142 (73.2 %) | 905 (58.0 %) | 2119 (48.9 %) |
| Antianemic preparations | 21287 (65.7 %) | 20274 (77.0 %) | 115 (59.3 %) | 536 (34.3 %) | 362 (8.4 %) |
| Drugs for functional gastrointestinal disorders | 18540 (57.2 %) | 16518 (62.8 %) | 116 (59.8 %) | 621 (39.8 %) | 1285 (29.6 %) |
| Vitamins | 13170 (40.6 %) | 12970 (49.3 %) | 43 (22.2 %) | 40 (2.6 %) | 117 (2.7 %) |
| Antibacterials for systemic use | 13152 (40.6 %) | 11534 (43.8 %) | 83 (42.8 %) | 431 (27.6 %) | 1104 (25.5 %) |
| Drugs for acid related disorders | 11966 (36.9 %) | 11061 (42.0 %) | 58 (29.9 %) | 310 (19.9 %) | 537 (12.4 %) |
| Gynecological antiinfectives and antiseptics | 9427 (29.1 %) | 8867 (33.7 %) | 48 (24.7 %) | 129 (8.3 %) | 383 (8.8 %) |
| Nasal preparations | 8613 (26.6 %) | 7914 (30.1 %) | 50 (25.8 %) | 181 (11.6 %) | 468 (10.8 %) |
| Antifungals for dermatological use | 7271 (22.4 %) | 6891 (26.2 %) | 33 (17.0 %) | 101 (6.5 %) | 246 (5.7 %) |
| Sex hormones and modulators of the genital system | 7084 (21.9 %) | 4999 (19.0 %) | 35 (18.0 %) | 392 (25.1 %) | 1658 (38.3 %) |
| Cough and cold preparations | 7003 (21.6 %) | 6503 (24.7 %) | 34 (17.5 %) | 138 (8.8 %) | 328 (7.6 %) |
| Antiseptics and disinfectants | 4761 (14.7 %) | 3818 (14.5 %) | 24 (12.4 %) | 242 (15.5 %) | 677 (15.6 %) |
| Contact laxatives | 4442 (13.7 %) | 4233 (16.1 %) | 31 (16.0 %) | 70 (4.5 %) | 108 (2.5 %) |
| Antiinflammatories and antirhumatics | 4355 (13.4 %) | 2757 (10.5 %) | 18 (9.3 %) | 297 (19.0 %) | 1283 (29.6 %) |
| Antiemetics and antinauseants | 3519 (10.9 %) | 3133 (11.9 %) | 26 (13.4 %) | 82 (5.3 %) | 278 (6.4 %) |
| Other gynecologicals | 3347 (10.3 %) | 2855 (10.8 %) | 24 (12.4 %) | 91 (5.8 %) | 377 (8.7 %) |
| Corticosteroids for systemic use | 3122 (9.6 %) | 2790 (10.6 %) | 17 (8.8 %) | 93 (6.0 %) | 222 (5.1 %) |
| Antihistamines for systemic use | 2978 (9.2 %) | 2532 (9.6 %) | 23 (11.9 %) | 104 (6.7 %) | 319 (7.4 %) |
| Stomatological preparations | 2803 (8.6 %) | 2592 (9.8 %) | 11 (5.7 %) | 57 (3.7 %) | 143 (3.3 %) |
| Immune sera and immunoglobulins | 2703 (8.3 %) | 2428 (9.2 %) | 11 (5.7 %) | 61 (3.9 %) | 203 (4.7 %) |
| Mineral supplements | 2614 (8.1 %) | 2512 (9.5 %) | 12 (6.2 %) | 32 (2.0 %) | 58 (1.3 %) |
| Homeopathic specialties | 2540 (7.8 %) | 2463 (9.4 %) | 11 (5.7 %) | 21 (1.3 %) | 45 (1.0 %) |
| Corticosteroids, dermatological preparations | 2504 (7.7 %) | 2285 (8.7 %) | 17 (8.8 %) | 49 (3.1 %) | 153 (3.5 %) |
| Drugs for obstructive airway diseases | 2443 (7.5 %) | 2202 (8.4 %) | 15 (7.7 %) | 69 (4.4 %) | 157 (3.6 %) |
| Psycholeptics | 2147 (6.6 %) | 1614 (6.1 %) | 33 (17.0 %) | 101 (6.5 %) | 399 (9.2 %) |
| Antithrombotic agents | 1927 (5.9 %) | 1831 (7.0 %) | 14 (7.2 %) | 45 (2.9 %) | 37 (0.9 %) |
| Emollients and protectives | 1921 (5.9 %) | 1790 (6.8 %) | 12 (6.2 %) | 19 (1.2 %) | 100 (2.3 %) |
| Ophthalmologicals | 1863 (5.7 %) | 1689 (6.4 %) | 15 (7.7 %) | 45 (2.9 %) | 114 (2.6 %) |
| Antibiotics and chemotherapeutics for dermatological use | 1654 (5.1 %) | 1498 (5.7 %) | 9 (4.6 %) | 42 (2.7 %) | 105 (2.4 %) |
| Vaccines | 1606 (5.0 %) | 1499 (5.7 %) | 4 (2.1 %) | 17 (1.1 %) | 86 (2.0 %) |
| Calcium channel blockers | 1392 (4.3 %) | 1364 (5.2 %) | 7 (3.6 %) | 6 (0.4 %) | 15 (0.3 %) |
| Topical products for joint and muscular pain | 1243 (3.8 %) | 1057 (4.0 %) | 8 (4.1 %) | 51 (3.3 %) | 127 (2.9 %) |
| Antidiarrheals, intestinal antiinflammatory/antiinfective agents | 1203 (3.7 %) | 1063 (4.0 %) | 7 (3.6 %) | 30 (1.9 %) | 103 (2.4 %) |
| Antiprotozoals | 1157 (3.6 %) | 1022 (3.9 %) | 8 (4.1 %) | 16 (1.0 %) | 111 (2.6 %) |
| Otologicals Peripheral vasodilators | 1052 (3.2 %) | 976 (3.7 %) | 5 (2.6 %) | 21 (1.3 %) | 50 (1.2 %) |
| Thyroid preparations | 993 (3.1 %) | 844 (3.2 %) | 10 (5.2 %) | 57 (3.7 %) | 82 (1.9 %) |
| Vasoprotective | 921 (2.8 %) | 896 (3.4 %) | 3 (1.5 %) | 7 (0.4 %) | 15 (0.3 %) |
| Anti-acne preparations | 859 (2.7 %) | 748 (2.8 %) | 4 (2.1 %) | 20 (1.3 %) | 87 (2.0 %) |
| Drugs used in diabetes | 789 (2.4 %) | 755 (2.9 %) | 3 (1.5 %) | 14 (0.9 %) | 17 (0.4 %) |
| Psychoanaleptics | 691 (2.1 %) | 424 (1.6 %) | 3 (1.5 %) | 47 (3.0 %) | 217 (5.0 %) |
| Blood substitutes and perfusion solutions | 548 (1.7 %) | 528 (2.0 %) | 4 (2.1 %) | 6 (0.4 %) | 10 (0.2 %) |
| Anesthetics | 534 (1.6 %) | 446 (1.7 %) | 2 (1.0 %) | 11 (0.7 %) | 75 (1.7 %) |
| Antivirals for systemic use | 512 (1.6 %) | 454 (1.7 %) | 0 (0%) | 15 (1.0 %) | 43 (1.0 %) |
| Antihemorrhagics | 487 (1.5 %) | 349 (1.3 %) | 0 (0%) | 20 (1.3 %) | 118 (2.7 %) |
| Other nervous system drugs | 443 (1.4 %) | 372 (1.4 %) | 2 (1.0 %) | 14 (0.9 %) | 55 (1.3 %) |
| Beta blocking agents | 404 (1.2 %) | 343 (1.3 %) | 7 (3.6 %) | 15 (1.0 %) | 39 (0.9 %) |
| Muscle relaxants | 343 (1.1 %) | 240 (0.9 %) | 0 (0%) | 17 (1.1 %) | 86 (2.0 %) |
| Antihypertensives | 257 (0.8 %) | 231 (0.9 %) | 5 (2.6 %) | 10 (0.6 %) | 11 (0.3 %) |
| Anthelmintics | 234 (0.7 %) | 200 (0.8 %) | 1 (0.5 %) | 6 (0.4 %) | 27 (0.6 %) |
| Peripheral vasodilators | 222 (0.7 %) | 215 (0.8 %) | 3 (1.5 %) | 2 (0.1 %) | 2 (0.0 %) |

**Note:** The sample size can vary given that some pregnancies did not use medication. A pregnancy can be counted more than once if a woman used more than one medications.

**Table C. Top 50 medication classes use in the year following the pregnancy (yes vs. no): stratified by pregnancy outcomes**

| **Top 50 medication classes use in the year following the pregnancy (yes vs. no**) | **All pregnancies 2010 - 2013 (n=34475)** | **Livebirths (n=26179)** | **Stillbirths (n=197)** | **Spontaneous abortions (n=1968)** | **Planned abortions (n=6131)** |
| --- | --- | --- | --- | --- | --- |
| Analgesics | 27644 (80.2 %) | 21173 (80.9 %) | 168 (85.3 %) | 1636 (83.1 %) | 4667 (76.1 %) |
| Antibacterials for systemic use | 19227 (55.8 %) | 14141 (54.0 %) | 123 (62.4 %) | 1197 (60.8 %) | 3766 (61.4 %) |
| Sex hormones and modulators of the genital system | 18544 (53.8 %) | 14003 (53.5 %) | 97 (49.2 %) | 977 (49.6 %) | 3467 (56.5 %) |
| Antiinflammatories and antirhumatics | 17313 (50.2 %) | 12769 (48.8 %) | 106 (53.8 %) | 943 (47.9 %) | 3495 (57.0 %) |
| Drugs for functional gastrointestinal disorders | 13640 (39.6 %) | 9804 (37.4 %) | 108 (54.8 %) | 1115 (56.7 %) | 2613 (42.6 %) |
| Antianemic preparations | 13309 (38.6 %) | 10827 (41.4 %) | 125 (63.5 %) | 1120 (56.9 %) | 1237 (20.2 %) |
| Nasal preparations | 11858 (34.4 %) | 8906 (34.0 %) | 58 (29.4 %) | 742 (37.7 %) | 2152 (35.1 %) |
| Drugs for acid related disorders | 9074 (26.3 %) | 6400 (24.4 %) | 72 (36.5 %) | 787 (40.0 %) | 1815 (29.6 %) |
| Cough and cold preparations | 8744 (25.4 %) | 6585 (25.2 %) | 56 (28.4 %) | 546 (27.7 %) | 1557 (25.4 %) |
| Other gynecologicals | 8105 (23.5 %) | 7091 (27.1 %) | 55 (27.9 %) | 195 (9.9 %) | 764 (12.5 %) |
| Corticosteroids for systemic use | 7898 (22.9 %) | 5925 (22.6 %) | 44 (22.3 %) | 479 (24.3 %) | 1450 (23.7 %) |
| Antihistamines for systemic use | 7858 (22.8 %) | 5778 (22.1 %) | 39 (19.8 %) | 469 (23.8 %) | 1572 (25.6 %) |
| Antiseptics and disinfectants | 7580 (22.0 %) | 5608 (21.4 %) | 50 (25.4 %) | 528 (26.8 %) | 1394 (22.7 %) |
| Antifungals for dermatological use | 6596 (19.1 %) | 4885 (18.7 %) | 48 (24.4 %) | 467 (23.7 %) | 1196 (19.5 %) |
| Gynecological antiinfectives and antiseptics | 6366 (18.5 %) | 4360 (16.7 %) | 64 (32.5 %) | 535 (27.2 %) | 1407 (22.9 %) |
| Vaccines | 5939 (17.2 %) | 5237 (20.0 %) | 23 (11.7 %) | 177 (9.0 %) | 502 (8.2 %) |
| Vitamins | 5645 (16.4 %) | 4431 (16.9 %) | 42 (21.3 %) | 480 (24.4 %) | 692 (11.3 %) |
| Stomatological preparations | 5236 (15.2 %) | 4020 (15.4 %) | 26 (13.2 %) | 257 (13.1 %) | 933 (15.2 %) |
| Topical products for joint and muscular pain | 4797 (13.9 %) | 3559 (13.6 %) | 37 (18.8 %) | 265 (13.5 %) | 936 (15.3 %) |
| Corticosteroids, dermatological preparations | 4770 (13.8 %) | 3654 (14.0 %) | 31 (15.7 %) | 259 (13.2 %) | 826 (13.5 %) |
| Psycholeptics | 4670 (13.5 %) | 2879 (11.0 %) | 76 (38.6 %) | 369 (18.8 %) | 1346 (22.0 %) |
| Ophthalmologicals | 4658 (13.5 %) | 3600 (13.8 %) | 34 (17.3 %) | 248 (12.6 %) | 776 (12.7 %) |
| Antidiarrheals, intestinal antiinflammatory/antiinfective agents | 3737 (10.8 %) | 2877 (11.0 %) | 18 (9.1 %) | 180 (9.1 %) | 662 (10.8 %) |
| Drugs for obstructive airway diseases | 3674 (10.7 %) | 2660 (10.2 %) | 15 (7.6 %) | 253 (12.9 %) | 746 (12.2 %) |
| Contact laxatives | 3456 (10.0 %) | 2672 (10.2 %) | 25 (12.7 %) | 255 (13.0 %) | 504 (8.2 %) |
| Antithrombotic agents | 3266 (9.5 %) | 2821 (10.8 %) | 35 (17.8 %) | 198 (10.1 %) | 212 (3.5 %) |
| Antibiotics and chemotherapeutics for dermatological use | 3264 (9.5 %) | 2485 (9.5 %) | 17 (8.6 %) | 193 (9.8 %) | 569 (9.3 %) |
| Mineral supplements | 2993 (8.7 %) | 2729 (10.4 %) | 15 (7.6 %) | 92 (4.7 %) | 157 (2.6 %) |
| Anesthetics | 2544 (7.4 %) | 2069 (7.9 %) | 11 (5.6 %) | 60 (3.0 %) | 404 (6.6 %) |
| Emollients and protectives | 2544 (7.4 %) | 1951 (7.5 %) | 15 (7.6 %) | 144 (7.3 %) | 434 (7.1 %) |
| Antiemetics and antinauseants | 2448 (7.1 %) | 1647 (6.3 %) | 16 (8.1 %) | 191 (9.7 %) | 594 (9.7 %) |
| Muscle relaxants | 2247 (6.5 %) | 1493 (5.7 %) | 13 (6.6 %) | 141 (7.2 %) | 600 (9.8 %) |
| Psychoanaleptics | 2017 (5.9 %) | 1255 (4.8 %) | 23 (11.7 %) | 148 (7.5 %) | 591 (9.6 %) |
| Otologicals | 2004 (5.8 %) | 1534 (5.9 %) | 4 (2.0 %) | 110 (5.6 %) | 356 (5.8 %) |
| Antihemorrhagics | 1911 (5.5 %) | 1527 (5.8 %) | 4 (2.0 %) | 68 (3.5 %) | 312 (5.1 %) |
| Homeopathic specialties | 1691 (4.9 %) | 1351 (5.2 %) | 14 (7.1 %) | 129 (6.6 %) | 197 (3.2 %) |
| Anti-acne preparations | 1567 (4.5 %) | 1019 (3.9 %) | 6 (3.0 %) | 100 (5.1 %) | 442 (7.2 %) |
| Antiprotozoals | 1542 (4.5 %) | 931 (3.6 %) | 12 (6.1 %) | 133 (6.8 %) | 466 (7.6 %) |
| Contrast media | 1226 (3.6 %) | 774 (3.0 %) | 17 (8.6 %) | 170 (8.6 %) | 265 (4.3 %) |
| Thyroid preparations | 1153 (3.3 %) | 918 (3.5 %) | 9 (4.6 %) | 85 (4.3 %) | 141 (2.3 %) |
| Antivirals for systemic use | 960 (2.8 %) | 681 (2.6 %) | 3 (1.5 %) | 73 (3.7 %) | 203 (3.3 %) |
| Other nervous system drugs | 882 (2.6 %) | 622 (2.4 %) | 4 (2.0 %) | 71 (3.6 %) | 185 (3.0 %) |
| Anthelmintics | 844 (2.4 %) | 598 (2.3 %) | 4 (2.0 %) | 41 (2.1 %) | 201 (3.3 %) |
| Vasoprotective | 759 (2.2 %) | 669 (2.6 %) | 2 (1.0 %) | 24 (1.2 %) | 64 (1.0 %) |
| Antimycotics for systemic use | 639 (1.9 %) | 475 (1.8 %) | 3 (1.5 %) | 43 (2.2 %) | 118 (1.9 %) |
| Blood substitutes and perfusion solutions | 626 (1.8 %) | 479 (1.8 %) | 5 (2.5 %) | 40 (2.0 %) | 102 (1.7 %) |
| Beta blocking agents | 577 (1.7 %) | 429 (1.6 %) | 6 (3.0 %) | 39 (2.0 %) | 103 (1.7 %) |
| Calcium channel blockers | 519 (1.5 %) | 402 (1.5 %) | 15 (7.6 %) | 38 (1.9 %) | 64 (1.0 %) |
| Antiepileptics | 425 (1.2 %) | 272 (1.0 %) | 2 (1.0 %) | 30 (1.5 %) | 121 (2.0 %) |
| Immune sera and immunoglobulins | 401 (1.2 %) | 110 (0.4 %) | 7 (3.6 %) | 89 (4.5 %) | 195 (3.2 %) |

**Note:** The sample size can vary given that some pregnancies did not use medication. A pregnancy can be counted more than once if a woman used more than one medications.

**Table D.** Top 50 medication classes use during the first trimester of pregnancy (yes vs. no): stratified by pregnancy outcomes

| **Top 50 medication classes use during the first trimester of pregnancy (yes vs. no)** | **All pregnancies 2010 - 2013 (n=27554)** | **Livebirths (n=21524)** | **Stillbirths (n=169)** | **Spontaneous abortions (n=1553)** | **Planned abortions (n=4308)** |
| --- | --- | --- | --- | --- | --- |
| Analgesics | 14508 (52.7 %) | 11431 (53.1 %) | 97 (57.4 %) | 892 (57.4 %) | 2088 (48.5 %) |
| Drugs for functional gastrointestinal disorders | 12239 (44.4 %) | 10278 (47.8 %) | 85 (50.3 %) | 605 (39.0 %) | 1271 (29.5 %) |
| Antianemic preparations | 9787 (35.5 %) | 8847 (41.1 %) | 65 (38.5 %) | 521 (33.5 %) | 354 (8.2 %) |
| Antibacterials for systemic use | 7240 (26.3 %) | 5691 (26.4 %) | 50 (29.6 %) | 417 (26.9 %) | 1082 (25.1 %) |
| Drugs for acid related disorders | 5048 (18.3 %) | 4177 (19.4 %) | 39 (23.1 %) | 305 (19.6 %) | 527 (12.2 %) |
| Nasal preparations | 4752 (17.2 %) | 4086 (19.0 %) | 31 (18.3 %) | 179 (11.5 %) | 456 (10.6 %) |
| Sex hormones and modulators of the genital system | 4398 (16.0 %) | 2344 (10.9 %) | 22 (13.0 %) | 384 (24.7 %) | 1648 (38.3 %) |
| Gynecological antiinfectives and antiseptics | 3756 (13.6 %) | 3233 (15.0 %) | 25 (14.8 %) | 119 (7.7 %) | 379 (8.8 %) |
| Cough and cold preparations | 3552 (12.9 %) | 3078 (14.3 %) | 19 (11.2 %) | 136 (8.8 %) | 319 (7.4 %) |
| Antifungals for dermatological use | 2982 (10.8 %) | 2620 (12.2 %) | 20 (11.8 %) | 98 (6.3 %) | 244 (5.7 %) |
| Antiemetics and antinauseants | 2869 (10.4 %) | 2489 (11.6 %) | 21 (12.4 %) | 81 (5.2 %) | 278 (6.5 %) |
| Antiinflammatories and antirhumatics | 2771 (10.1 %) | 1204 (5.6 %) | 10 (5.9 %) | 287 (18.5 %) | 1270 (29.5 %) |
| Antiseptics and disinfectants | 2055 (7.5 %) | 1136 (5.3 %) | 11 (6.5 %) | 235 (15.1 %) | 673 (15.6 %) |
| Contact laxatives | 2037 (7.4 %) | 1853 (8.6 %) | 13 (7.7 %) | 66 (4.2 %) | 105 (2.4 %) |
| Antihistamines for systemic use | 1714 (6.2 %) | 1289 (6.0 %) | 16 (9.5 %) | 101 (6.5 %) | 308 (7.1 %) |
| Vitamins | 1489 (5.4 %) | 1325 (6.2 %) | 9 (5.3 %) | 39 (2.5 %) | 116 (2.7 %) |
| Corticosteroids for systemic use | 1488 (5.4 %) | 1172 (5.4 %) | 9 (5.3 %) | 91 (5.9 %) | 216 (5.0 %) |
| Drugs for obstructive airway diseases | 1374 (5.0 %) | 1149 (5.3 %) | 6 (3.6 %) | 67 (4.3 %) | 152 (3.5 %) |
| Psycholeptics | 1307 (4.7 %) | 816 (3.8 %) | 10 (5.9 %) | 97 (6.2 %) | 384 (8.9 %) |
| Corticosteroids, dermatological preparations | 1213 (4.4 %) | 1007 (4.7 %) | 10 (5.9 %) | 47 (3.0 %) | 149 (3.5 %) |
| Stomatological preparations | 1205 (4.4 %) | 1006 (4.7 %) | 6 (3.6 %) | 55 (3.5 %) | 138 (3.2 %) |
| Ophthalmologicals | 917 (3.3 %) | 757 (3.5 %) | 9 (5.3 %) | 45 (2.9 %) | 106 (2.5 %) |
| Homeopathic specialties | 831 (3.0 %) | 762 (3.5 %) | 5 (3.0 %) | 21 (1.4 %) | 43 (1.0 %) |
| Thyroid preparations | 824 (3.0 %) | 678 (3.1 %) | 7 (4.1 %) | 57 (3.7 %) | 82 (1.9 %) |
| Emollients and protectives | 808 (2.9 %) | 685 (3.2 %) | 6 (3.6 %) | 19 (1.2 %) | 98 (2.3 %) |
| Antibiotics and chemotherapeutics for dermatological use | 806 (2.9 %) | 656 (3.0 %) | 5 (3.0 %) | 42 (2.7 %) | 103 (2.4 %) |
| Topical products for joint and muscular pain | 760 (2.8 %) | 583 (2.7 %) | 4 (2.4 %) | 49 (3.2 %) | 124 (2.9 %) |
| Mineral supplements | 724 (2.6 %) | 630 (2.9 %) | 5 (3.0 %) | 31 (2.0 %) | 58 (1.3 %) |
| Antidiarrheals, intestinal antiinflammatory/antiinfective agents | 702 (2.5 %) | 569 (2.6 %) | 3 (1.8 %) | 29 (1.9 %) | 101 (2.3 %) |
| Psychoanaleptics | 617 (2.2 %) | 354 (1.6 %) | 3 (1.8 %) | 46 (3.0 %) | 214 (5.0 %) |
| Anti-acne preparations | 546 (2.0 %) | 437 (2.0 %) | 3 (1.8 %) | 20 (1.3 %) | 86 (2.0 %) |
| Immune sera and immunoglobulins | 541 (2.0 %) | 275 (1.3 %) | 3 (1.8 %) | 60 (3.9 %) | 203 (4.7 %) |
| Other gynecologicals | 540 (2.0 %) | 90 (0.4 %) | 1 (0.6 %) | 83 (5.3 %) | 366 (8.5 %) |
| Otologicals | 460 (1.7 %) | 391 (1.8 %) | 0 (0%) | 21 (1.4 %) | 48 (1.1 %) |
| Antithrombotic agents | 416 (1.5 %) | 334 (1.6 %) | 2 (1.2 %) | 44 (2.8 %) | 36 (0.8 %) |
| Antiprotozoals | 402 (1.5 %) | 277 (1.3 %) | 2 (1.2 %) | 14 (0.9 %) | 109 (2.5 %) |
| Vaccines | 338 (1.2 %) | 234 (1.1 %) | 3 (1.8 %) | 17 (1.1 %) | 84 (1.9 %) |
| Other nervous system drugs | 289 (1.0 %) | 220 (1.0 %) | 0 (0%) | 14 (0.9 %) | 55 (1.3 %) |
| Muscle relaxants | 265 (1.0 %) | 166 (0.8 %) | 0 (0%) | 15 (1.0 %) | 84 (1.9 %) |
| Beta blocking agents | 244 (0.9 %) | 188 (0.9 %) | 4 (2.4 %) | 13 (0.8 %) | 39 (0.9 %) |
| Antivirals for systemic use | 223 (0.8 %) | 166 (0.8 %) | 0 (0%) | 15 (1.0 %) | 42 (1.0 %) |
| Drugs used in diabetes | 207 (0.8 %) | 175 (0.8 %) | 3 (1.8 %) | 13 (0.8 %) | 16 (0.4 %) |
| Anesthetics | 197 (0.7 %) | 109 (0.5 %) | 2 (1.2 %) | 11 (0.7 %) | 75 (1.7 %) |
| Antiepileptics | 181 (0.7 %) | 115 (0.5 %) | 3 (1.8 %) | 17 (1.1 %) | 46 (1.1 %) |
| Vasoprotective | 179 (0.6 %) | 157 (0.7 %) | 0 (0%) | 7 (0.5 %) | 15 (0.3 %) |
| Antihemorrhagics | 153 (0.6 %) | 16 (0.1 %) | 0 (0%) | 20 (1.3 %) | 117 (2.7 %) |
| Anthelmintics | 123 (0.4 %) | 90 (0.4 %) | 1 (0.6 %) | 6 (0.4 %) | 26 (0.6 %) |
| Calcium channel blockers | 100 (0.4 %) | 81 (0.4 %) | 1 (0.6 %) | 4 (0.3 %) | 14 (0.3 %) |
| Blood substitutes and perfusion solutions | 99 (0.4 %) | 83 (0.4 %) | 0 (0%) | 6 (0.4 %) | 10 (0.2 %) |
| Antihypertensives | 94 (0.3 %) | 70 (0.3 %) | 3 (1.8 %) | 10 (0.6 %) | 11 (0.3 %) |

**Note:** The sample size can vary given that some pregnancies did not use medication. A pregnancy can be counted more than once if a woman used more than one medications.

**Table E.** Top 50 medication classes use during the second trimester of pregnancy (yes vs. no): stratified by pregnancy outcomes

| **Top 50 medication classes use during the second trimester of pregnancy (yes vs. no)** | **All pregnancies 2010 - 2013 (n=22416)** | **Livebirths (n=22180)** | **Stillbirths (n=161)** | **Spontaneous abortion (n=43)** | **Planned abortion (n=32)** |
| --- | --- | --- | --- | --- | --- |
| Antianemic preparations | 10424 (46.5 %) | 10340 (46.6 %) | 60 (37.3 %) | 18 (41.9 %) | 6 (18.8 %) |
| Analgesics | 9998 (44.6 %) | 9882 (44.6 %) | 74 (46.0 %) | 22 (51.2 %) | 20 (62.5 %) |
| Drugs for functional gastrointestinal disorders | 7157 (31.9 %) | 7073 (31.9 %) | 55 (34.2 %) | 18 (41.9 %) | 11 (34.4 %) |
| Vitamins | 7074 (31.6 %) | 7044 (31.8 %) | 29 (18.0 %) | 1 (2.3 %) | 0 (0%) |
| Antibacterials for systemic use | 5221 (23.3 %) | 5161 (23.3 %) | 40 (24.8 %) | 16 (37.2 %) | 4 (12.5 %) |
| Drugs for acid related disorders | 5172 (23.1 %) | 5137 (23.2 %) | 27 (16.8 %) | 6 (14.0 %) | 2 (6.3 %) |
| Gynecological antiinfectives and antiseptics | 4091 (18.3 %) | 4060 (18.3 %) | 22 (13.7 %) | 8 (18.6 %) | 1 (3.1 %) |
| Nasal preparations | 3474 (15.5 %) | 3446 (15.5 %) | 25 (15.5 %) | 1 (2.3 %) | 2 (6.3 %) |
| Antifungals for dermatological use | 3100 (13.8 %) | 3084 (13.9 %) | 14 (8.7 %) | 1 (2.3 %) | 1 (3.1 %) |
| Cough and cold preparations | 2755 (12.3 %) | 2738 (12.3 %) | 14 (8.7 %) | 1 (2.3 %) | 2 (6.3 %) |
| Contact laxatives | 1553 (6.9 %) | 1536 (6.9 %) | 14 (8.7 %) | 2 (4.7 %) | 1 (3.1 %) |
| Immune sera and immunoglobulins | 1353 (6.0 %) | 1346 (6.1 %) | 6 (3.7 %) | 1 (2.3 %) | 0 (0%) |
| Corticosteroids for systemic use | 1089 (4.9 %) | 1079 (4.9 %) | 8 (5.0 %) | 2 (4.7 %) | 0 (0%) |
| Stomatological preparations | 1082 (4.8 %) | 1072 (4.8 %) | 7 (4.3 %) | 2 (4.7 %) | 1 (3.1 %) |
| Drugs for obstructive airway diseases | 1075 (4.8 %) | 1066 (4.8 %) | 9 (5.6 %) | 0 (0%) | 0 (0%) |
| Antiseptics and disinfectants | 1066 (4.8 %) | 1050 (4.7 %) | 10 (6.2 %) | 4 (9.3 %) | 2 (6.3 %) |
| Antihistamines for systemic use | 1024 (4.6 %) | 1015 (4.6 %) | 9 (5.6 %) | 0 (0%) | 0 (0%) |
| Mineral supplements | 883 (3.9 %) | 875 (3.9 %) | 6 (3.7 %) | 2 (4.7 %) | 0 (0%) |
| Corticosteroids, dermatological preparations | 875 (3.9 %) | 867 (3.9 %) | 7 (4.3 %) | 1 (2.3 %) | 0 (0%) |
| Homeopathic specialties | 870 (3.9 %) | 862 (3.9 %) | 6 (3.7 %) | 0 (0%) | 2 (6.3 %) |
| Emollients and protectives | 763 (3.4 %) | 753 (3.4 %) | 10 (6.2 %) | 0 (0%) | 0 (0%) |
| Thyroid preparations | 735 (3.3 %) | 726 (3.3 %) | 9 (5.6 %) | 0 (0%) | 0 (0%) |
| Ophthalmologicals | 624 (2.8 %) | 619 (2.8 %) | 5 (3.1 %) | 0 (0%) | 0 (0%) |
| Psycholeptics | 624 (2.8 %) | 595 (2.7 %) | 18 (11.2 %) | 4 (9.3 %) | 7 (21.9 %) |
| Antiemetics and antinauseants | 557 (2.5 %) | 552 (2.5 %) | 5 (3.1 %) | 0 (0%) | 0 (0%) |
| Antibiotics and chemotherapeutics for dermatological use | 508 (2.3 %) | 504 (2.3 %) | 4 (2.5 %) | 0 (0%) | 0 (0%) |
| Other gynecologicals | 401 (1.8 %) | 369 (1.7 %) | 13 (8.1 %) | 8 (18.6 %) | 11 (34.4 %) |
| Calcium channel blockers | 389 (1.7 %) | 386 (1.7 %) | 2 (1.2 %) | 1 (2.3 %) | 0 (0%) |
| Otologicals | 346 (1.5 %) | 341 (1.5 %) | 5 (3.1 %) | 0 (0%) | 0 (0%) |
| Drugs used in diabetes | 337 (1.5 %) | 334 (1.5 %) | 1 (0.6 %) | 1 (2.3 %) | 1 (3.1 %) |
| Antidiarrheals, intestinal antiinflammatory/antiinfective agents | 331 (1.5 %) | 328 (1.5 %) | 3 (1.9 %) | 0 (0%) | 0 (0%) |
| Antithrombotic agents | 328 (1.5 %) | 319 (1.4 %) | 5 (3.1 %) | 2 (4.7 %) | 2 (6.3 %) |
| Anti-acne preparations | 313 (1.4 %) | 312 (1.4 %) | 1 (0.6 %) | 0 (0%) | 0 (0%) |
| Topical products for joint and muscular pain | 311 (1.4 %) | 308 (1.4 %) | 3 (1.9 %) | 0 (0%) | 0 (0%) |
| Antiprotozoals | 308 (1.4 %) | 300 (1.4 %) | 4 (2.5 %) | 3 (7.0 %) | 1 (3.1 %) |
| Sex hormones and modulators of the genital system | 298 (1.3 %) | 286 (1.3 %) | 7 (4.3 %) | 3 (7.0 %) | 2 (6.3 %) |
| Antiinflammatories and antirhumatics | 269 (1.2 %) | 264 (1.2 %) | 2 (1.2 %) | 2 (4.7 %) | 1 (3.1 %) |
| Blood substitutes and perfusion solutions | 261 (1.2 %) | 257 (1.2 %) | 4 (2.5 %) | 0 (0%) | 0 (0%) |
| Vasoprotective | 242 (1.1 %) | 240 (1.1 %) | 2 (1.2 %) | 0 (0%) | 0 (0%) |
| Psychoanaleptics | 189 (0.8 %) | 188 (0.8 %) | 1 (0.6 %) | 0 (0%) | 0 (0%) |
| Beta blocking agents | 181 (0.8 %) | 175 (0.8 %) | 5 (3.1 %) | 0 (0%) | 1 (3.1 %) |
| Other nervous system drugs | 166 (0.7 %) | 164 (0.7 %) | 2 (1.2 %) | 0 (0%) | 0 (0%) |
| Antivirals for systemic use | 155 (0.7 %) | 155 (0.7 %) | 0 (0%) | 0 (0%) | 0 (0%) |
| Vaccines | 142 (0.6 %) | 140 (0.6 %) | 1 (0.6 %) | 0 (0%) | 1 (3.1 %) |
| Antihypertensives | 110 (0.5 %) | 106 (0.5 %) | 4 (2.5 %) | 0 (0%) | 0 (0%) |
| Anesthetics | 104 (0.5 %) | 104 (0.5 %) | 0 (0%) | 0 (0%) | 0 (0%) |
| Antiepileptics | 94 (0.4 %) | 92 (0.4 %) | 2 (1.2 %) | 0 (0%) | 0 (0%) |
| Anthelmintics | 68 (0.3 %) | 68 (0.3 %) | 0 (0%) | 0 (0%) | 0 (0%) |
| Muscle relaxants | 51 (0.2 %) | 51 (0.2 %) | 0 (0%) | 0 (0%) | 0 (0%) |
| Antimycotics for systemic use | 41 (0.2 %) | 41 (0.2 %) | 0 (0%) | 0 (0%) | 0 (0%) |

**Note:** The sample size can vary given that some pregnancies did not use medication. A pregnancy can be counted more than once if a woman used more than one medications.

**Table F.** Top 50 medication classes use during the third trimester of pregnancy (yes vs. no): stratified by pregnancy outcomes

| **Top 50 medication classes use during the third trimester of pregnancy (yes vs. no)** | **All pregnancies 2010 - 2013 (n=23932)** | **Livebirths (n=23861)** | **Stillbirths (n=71)** | **Spontaneous abortions  (n=.)** | **Planned abortions  (n=.)** |
| --- | --- | --- | --- | --- | --- |
| Antianemic preparations | 15170 (63.4 %) | 15129 (63.4 %) | 41 (57.7 %) | 0 (0%) | 0 (0%) |
| Analgesics | 11471 (47.9 %) | 11438 (47.9 %) | 33 (46.5 %) | 0 (0%) | 0 (0%) |
| Drugs for functional gastrointestinal disorders | 7094 (29.6 %) | 7079 (29.7 %) | 15 (21.1 %) | 0 (0%) | 0 (0%) |
| Drugs for acid related disorders | 6226 (26.0 %) | 6212 (26.0 %) | 14 (19.7 %) | 0 (0%) | 0 (0%) |
| Vitamins | 5974 (25.0 %) | 5966 (25.0 %) | 8 (11.3 %) | 0 (0%) | 0 (0%) |
| Gynecological antiinfectives and antiseptics | 4602 (19.2 %) | 4595 (19.3 %) | 7 (9.9 %) | 0 (0%) | 0 (0%) |
| Antibacterials for systemic use | 4505 (18.8 %) | 4491 (18.8 %) | 14 (19.7 %) | 0 (0%) | 0 (0%) |
| Antifungals for dermatological use | 2921 (12.2 %) | 2917 (12.2 %) | 4 (5.6 %) | 0 (0%) | 0 (0%) |
| Sex hormones and modulators of the genital system | 2669 (11.2 %) | 2661 (11.2 %) | 8 (11.3 %) | 0 (0%) | 0 (0%) |
| Other gynecologicals | 2555 (10.7 %) | 2544 (10.7 %) | 11 (15.5 %) | 0 (0%) | 0 (0%) |
| Nasal preparations | 2386 (10.0 %) | 2381 (10.0 %) | 5 (7.0 %) | 0 (0%) | 0 (0%) |
| Antiseptics and disinfectants | 2118 (8.9 %) | 2111 (8.8 %) | 7 (9.9 %) | 0 (0%) | 0 (0%) |
| Cough and cold preparations | 1910 (8.0 %) | 1906 (8.0 %) | 4 (5.6 %) | 0 (0%) | 0 (0%) |
| Contact laxatives | 1709 (7.1 %) | 1704 (7.1 %) | 5 (7.0 %) | 0 (0%) | 0 (0%) |
| Antithrombotic agents | 1601 (6.7 %) | 1592 (6.7 %) | 9 (12.7 %) | 0 (0%) | 0 (0%) |
| Mineral supplements | 1442 (6.0 %) | 1439 (6.0 %) | 3 (4.2 %) | 0 (0%) | 0 (0%) |
| Antiinflammatories and antirhumatics | 1376 (5.7 %) | 1371 (5.7 %) | 5 (7.0 %) | 0 (0%) | 0 (0%) |
| Homeopathic specialties | 1240 (5.2 %) | 1239 (5.2 %) | 1 (1.4 %) | 0 (0%) | 0 (0%) |
| Calcium channel blockers | 1154 (4.8 %) | 1149 (4.8 %) | 5 (7.0 %) | 0 (0%) | 0 (0%) |
| Vaccines | 1142 (4.8 %) | 1142 (4.8 %) | 0 (0%) | 0 (0%) | 0 (0%) |
| Immune sera and immunoglobulins | 1013 (4.2 %) | 1011 (4.2 %) | 2 (2.8 %) | 0 (0%) | 0 (0%) |
| Corticosteroids for systemic use | 961 (4.0 %) | 961 (4.0 %) | 0 (0%) | 0 (0%) | 0 (0%) |
| Drugs for obstructive airway diseases | 854 (3.6 %) | 851 (3.6 %) | 3 (4.2 %) | 0 (0%) | 0 (0%) |
| Emollients and protectives | 795 (3.3 %) | 795 (3.3 %) | 0 (0%) | 0 (0%) | 0 (0%) |
| Antihistamines for systemic use | 791 (3.3 %) | 790 (3.3 %) | 1 (1.4 %) | 0 (0%) | 0 (0%) |
| Stomatological preparations | 789 (3.3 %) | 788 (3.3 %) | 1 (1.4 %) | 0 (0%) | 0 (0%) |
| Thyroid preparations | 755 (3.2 %) | 750 (3.1 %) | 5 (7.0 %) | 0 (0%) | 0 (0%) |
| Corticosteroids, dermatological preparations | 732 (3.1 %) | 731 (3.1 %) | 1 (1.4 %) | 0 (0%) | 0 (0%) |
| Drugs used in diabetes | 633 (2.6 %) | 633 (2.7 %) | 0 (0%) | 0 (0%) | 0 (0%) |
| Psycholeptics | 616 (2.6 %) | 608 (2.5 %) | 8 (11.3 %) | 0 (0%) | 0 (0%) |
| Vasoprotective | 584 (2.4 %) | 583 (2.4 %) | 1 (1.4 %) | 0 (0%) | 0 (0%) |
| Antiprotozoals | 543 (2.3 %) | 541 (2.3 %) | 2 (2.8 %) | 0 (0%) | 0 (0%) |
| Ophthalmologicals | 499 (2.1 %) | 497 (2.1 %) | 2 (2.8 %) | 0 (0%) | 0 (0%) |
| Antibiotics and chemotherapeutics for dermatological use | 451 (1.9 %) | 451 (1.9 %) | 0 (0%) | 0 (0%) | 0 (0%) |
| Antihemorrhagics | 327 (1.4 %) | 327 (1.4 %) | 0 (0%) | 0 (0%) | 0 (0%) |
| Antiemetics and antinauseants | 313 (1.3 %) | 310 (1.3 %) | 3 (4.2 %) | 0 (0%) | 0 (0%) |
| Otologicals | 299 (1.2 %) | 299 (1.3 %) | 0 (0%) | 0 (0%) | 0 (0%) |
| Anesthetics | 267 (1.1 %) | 267 (1.1 %) | 0 (0%) | 0 (0%) | 0 (0%) |
| Antivirals for systemic use | 256 (1.1 %) | 256 (1.1 %) | 0 (0%) | 0 (0%) | 0 (0%) |
| Antidiarrheals, intestinal antiinflammatory/antiinfective agents | 248 (1.0 %) | 248 (1.0 %) | 0 (0%) | 0 (0%) | 0 (0%) |
| Topical products for joint and muscular pain | 220 (0.9 %) | 219 (0.9 %) | 1 (1.4 %) | 0 (0%) | 0 (0%) |
| Blood substitutes and perfusion solutions | 218 (0.9 %) | 218 (0.9 %) | 0 (0%) | 0 (0%) | 0 (0%) |
| Beta blocking agents | 209 (0.9 %) | 209 (0.9 %) | 0 (0%) | 0 (0%) | 0 (0%) |
| Peripheral vasodilators | 202 (0.8 %) | 201 (0.8 %) | 1 (1.4 %) | 0 (0%) | 0 (0%) |
| Antihypertensives | 192 (0.8 %) | 192 (0.8 %) | 0 (0%) | 0 (0%) | 0 (0%) |
| Psychoanaleptics | 165 (0.7 %) | 164 (0.7 %) | 1 (1.4 %) | 0 (0%) | 0 (0%) |
| Anti-acne preparations | 135 (0.6 %) | 135 (0.6 %) | 0 (0%) | 0 (0%) | 0 (0%) |
| Other nervous system drugs | 115 (0.5 %) | 115 (0.5 %) | 0 (0%) | 0 (0%) | 0 (0%) |
| Antiepileptics | 93 (0.4 %) | 92 (0.4 %) | 1 (1.4 %) | 0 (0%) | 0 (0%) |
| Hepatic and biliary tract therapeutics | 74 (0.3 %) | 74 (0.3 %) | 0 (0%) | 0 (0%) | 0 (0%) |

**Note:** The sample size can vary given that some pregnancies did not use medication. A pregnancy can be counted more than once if a woman used more than one medications.

**Table G. Top 50 medication use in the year prior to pregnancy (yes vs. no): stratified by pregnancy outcomes**

| **Top 50 medication use in the year prior to pregnancy (yes vs. no**) | **All pregnancies 2010 - 2013 (n=32839)** | **Livebirths (n=24877)** | **Stillbirths (n=189)** | **Spontaneous abortions (n=1894)** | **Planned abortions (n=5879)** |
| --- | --- | --- | --- | --- | --- |
| Acetaminophen/paracetamol | 22521 (68.6 %) | 17016 (68.4 %) | 133 (70.4 %) | 1357 (71.6 %) | 4015 (68.3 %) |
| Ibuprofen | 9557 (29.1 %) | 7155 (28.8 %) | 53 (28.0 %) | 584 (30.8 %) | 1765 (30.0 %) |
| Phloroglucinol | 9416 (28.7 %) | 7175 (28.8 %) | 57 (30.2 %) | 601 (31.7 %) | 1583 (26.9 %) |
| Levonorgestrel and estrogen | 8725 (26.6 %) | 6614 (26.6 %) | 41 (21.7 %) | 452 (23.9 %) | 1618 (27.5 %) |
| Amoxicillin | 7839 (23.9 %) | 5919 (23.8 %) | 43 (22.8 %) | 441 (23.3 %) | 1436 (24.4 %) |
| Folic acid | 6556 (20.0 %) | 5777 (23.2 %) | 34 (18.0 %) | 412 (21.8 %) | 333 (5.7 %) |
| Prednisolone | 6107 (18.6 %) | 4610 (18.5 %) | 29 (15.3 %) | 368 (19.4 %) | 1100 (18.7 %) |
| Diclofenac | 5169 (15.7 %) | 3850 (15.5 %) | 35 (18.5 %) | 354 (18.7 %) | 930 (15.8 %) |
| Prednisolone in combination | 4562 (13.9 %) | 3476 (14.0 %) | 24 (12.7 %) | 270 (14.3 %) | 792 (13.5 %) |
| Ketoprofen | 4527 (13.8 %) | 3368 (13.5 %) | 27 (14.3 %) | 299 (15.8 %) | 833 (14.2 %) |
| Ferrous sulfate | 4412 (13.4 %) | 3221 (12.9 %) | 22 (11.6 %) | 258 (13.6 %) | 911 (15.5 %) |
| Amoxicillin and enzyme inhibitor | 4372 (13.3 %) | 3211 (12.9 %) | 27 (14.3 %) | 288 (15.2 %) | 846 (14.4 %) |
| Domperidone | 4280 (13.0 %) | 3287 (13.2 %) | 24 (12.7 %) | 233 (12.3 %) | 736 (12.5 %) |
| Tuaminoheptane | 3684 (11.2 %) | 2807 (11.3 %) | 11 (5.8 %) | 251 (13.3 %) | 615 (10.5 %) |
| Betamethasone | 3644 (11.1 %) | 2760 (11.1 %) | 26 (13.8 %) | 214 (11.3 %) | 644 (11.0 %) |
| Helicidine | 3531 (10.8 %) | 2669 (10.7 %) | 21 (11.1 %) | 223 (11.8 %) | 618 (10.5 %) |
| Stomatological preparations (antiinfectives and antiseptics for local oral treatment) | 3384 (10.3 %) | 2484 (10.0 %) | 27 (14.3 %) | 168 (8.9 %) | 705 (12.0 %) |
| Desloratadine | 3241 (9.9 %) | 2456 (9.9 %) | 16 (8.5 %) | 190 (10.0 %) | 579 (9.8 %) |
| Tixocortol | 3132 (9.5 %) | 2373 (9.5 %) | 14 (7.4 %) | 192 (10.1 %) | 553 (9.4 %) |
| Colecalciferol | 2953 (9.0 %) | 2190 (8.8 %) | 17 (9.0 %) | 190 (10.0 %) | 556 (9.5 %) |
| Pholcodine | 2899 (8.8 %) | 2205 (8.9 %) | 16 (8.5 %) | 159 (8.4 %) | 519 (8.8 %) |
| Codeine in combination | 2892 (8.8 %) | 2115 (8.5 %) | 16 (8.5 %) | 178 (9.4 %) | 583 (9.9 %) |
| Econazole | 2876 (8.8 %) | 2093 (8.4 %) | 21 (11.1 %) | 180 (9.5 %) | 582 (9.9 %) |
| Omeprazole | 2805 (8.5 %) | 2046 (8.2 %) | 20 (10.6 %) | 192 (10.1 %) | 547 (9.3 %) |
| Other drugs for acid related disorders | 2670 (8.1 %) | 2051 (8.2 %) | 10 (5.3 %) | 149 (7.9 %) | 460 (7.8 %) |
| Chlorhexidine in combination | 2631 (8.0 %) | 1894 (7.6 %) | 16 (8.5 %) | 155 (8.2 %) | 566 (9.6 %) |
| Oxomemazine | 2629 (8.0 %) | 1939 (7.8 %) | 10 (5.3 %) | 159 (8.4 %) | 521 (8.9 %) |
| Metopimazine | 2612 (8.0 %) | 1993 (8.0 %) | 17 (9.0 %) | 150 (7.9 %) | 452 (7.7 %) |
| Cefpodoxime | 2584 (7.9 %) | 1976 (7.9 %) | 7 (3.7 %) | 149 (7.9 %) | 452 (7.7 %) |
| Povidone-iodine | 2491 (7.6 %) | 1822 (7.3 %) | 19 (10.1 %) | 156 (8.2 %) | 494 (8.4 %) |
| Acid tiaprofenic | 2470 (7.5 %) | 1843 (7.4 %) | 15 (7.9 %) | 139 (7.3 %) | 473 (8.0 %) |
| Chlorhexidine | 2427 (7.4 %) | 1790 (7.2 %) | 16 (8.5 %) | 150 (7.9 %) | 471 (8.0 %) |
| Sertaconazole | 2397 (7.3 %) | 1807 (7.3 %) | 16 (8.5 %) | 146 (7.7 %) | 428 (7.3 %) |
| Racecadotril | 2306 (7.0 %) | 1796 (7.2 %) | 12 (6.3 %) | 121 (6.4 %) | 377 (6.4 %) |
| Tramadol in combination | 2296 (7.0 %) | 1664 (6.7 %) | 11 (5.8 %) | 156 (8.2 %) | 465 (7.9 %) |
| Salbutamol | 2264 (6.9 %) | 1664 (6.7 %) | 10 (5.3 %) | 142 (7.5 %) | 448 (7.6 %) |
| Loperamide | 2162 (6.6 %) | 1673 (6.7 %) | 11 (5.8 %) | 127 (6.7 %) | 351 (6.0 %) |
| Soft paraffin and fat products | 2162 (6.6 %) | 1581 (6.4 %) | 10 (5.3 %) | 129 (6.8 %) | 442 (7.5 %) |
| Fosfomycin | 2123 (6.5 %) | 1591 (6.4 %) | 11 (5.8 %) | 132 (7.0 %) | 389 (6.6 %) |
| Whooping cough, tetanus, diphtheria and polio | 2117 (6.4 %) | 1627 (6.5 %) | 17 (9.0 %) | 122 (6.4 %) | 351 (6.0 %) |
| Tetrazepam | 1998 (6.1 %) | 1503 (6.0 %) | 15 (7.9 %) | 141 (7.4 %) | 339 (5.8 %) |
| Desogestrel and estrogen | 1997 (6.1 %) | 1516 (6.1 %) | 10 (5.3 %) | 119 (6.3 %) | 352 (6.0 %) |
| Flurbiprofen | 1926 (5.9 %) | 1476 (5.9 %) | 11 (5.8 %) | 117 (6.2 %) | 322 (5.5 %) |
| Dextromethorphan | 1896 (5.8 %) | 1447 (5.8 %) | 11 (5.8 %) | 110 (5.8 %) | 328 (5.6 %) |
| Antitussive combinations | 1858 (5.7 %) | 1389 (5.6 %) | 12 (6.3 %) | 119 (6.3 %) | 338 (5.7 %) |
| Trimebutine | 1848 (5.6 %) | 1419 (5.7 %) | 7 (3.7 %) | 105 (5.5 %) | 317 (5.4 %) |
| Spiramycin combination with other antibacterials | 1836 (5.6 %) | 1311 (5.3 %) | 10 (5.3 %) | 113 (6.0 %) | 402 (6.8 %) |
| Progesterone | 1816 (5.5 %) | 1537 (6.2 %) | 16 (8.5 %) | 142 (7.5 %) | 121 (2.1 %) |
| Acid fusidic | 1802 (5.5 %) | 1289 (5.2 %) | 12 (6.3 %) | 121 (6.4 %) | 380 (6.5 %) |
| Esomeprazole | 1787 (5.4 %) | 1317 (5.3 %) | 21 (11.1 %) | 112 (5.9 %) | 337 (5.7 %) |

**Note:** The sample size can vary given that some pregnancies did not use medication. A pregnancy can be counted more than once if a woman used more than one medications.

**Table H.** Top 50 medication use during pregnancy (yes vs. no): stratified by pregnancy outcomes

| **Top 50 medication use during pregnancy (yes vs. no)** | **All pregnancies 2010 - 2013 (n=32408)** | **Livebirths (n=26319)** | **Stillbirths (n=194)** | **Spontaneous abortions (n=1561)** | **Planned abortions (n=4334)** |
| --- | --- | --- | --- | --- | --- |
| Acetaminophen/paracetamol | 21760 (67.1 %) | 19183 (72.9 %) | 137 (70.6 %) | 825 (52.9 %) | 1615 (37.3 %) |
| Ferrous sulfate | 18143 (56.0 %) | 17667 (67.1 %) | 86 (44.3 %) | 206 (13.2 %) | 184 (4.2 %) |
| Phloroglucinol | 15550 (48.0 %) | 14044 (53.4 %) | 99 (51.0 %) | 507 (32.5 %) | 900 (20.8 %) |
| Colecalciferol | 12993 (40.1 %) | 12809 (48.7 %) | 40 (20.6 %) | 38 (2.4 %) | 106 (2.4 %) |
| Folic acid | 8263 (25.5 %) | 7646 (29.1 %) | 52 (26.8 %) | 379 (24.3 %) | 186 (4.3 %) |
| Amoxicillin | 7252 (22.4 %) | 6776 (25.7 %) | 49 (25.3 %) | 149 (9.5 %) | 278 (6.4 %) |
| Alginic acid in combination with antacids | 6482 (20.0 %) | 6330 (24.1 %) | 32 (16.5 %) | 52 (3.3 %) | 68 (1.6 %) |
| Helicidine | 5429 (16.8 %) | 5193 (19.7 %) | 27 (13.9 %) | 79 (5.1 %) | 130 (3.0 %) |
| Sertaconazole | 4589 (14.2 %) | 4407 (16.7 %) | 22 (11.3 %) | 46 (2.9 %) | 114 (2.6 %) |
| Tixocortol | 4573 (14.1 %) | 4363 (16.6 %) | 23 (11.9 %) | 71 (4.5 %) | 116 (2.7 %) |
| Metoclopramide | 4555 (14.1 %) | 4182 (15.9 %) | 32 (16.5 %) | 91 (5.8 %) | 250 (5.8 %) |
| Econazole | 4414 (13.6 %) | 4223 (16.0 %) | 18 (9.3 %) | 54 (3.5 %) | 119 (2.7 %) |
| Domperidone | 4212 (13.0 %) | 3779 (14.4 %) | 30 (15.5 %) | 96 (6.1 %) | 307 (7.1 %) |
| Omeprazole | 3684 (11.4 %) | 3513 (13.3 %) | 19 (9.8 %) | 50 (3.2 %) | 102 (2.4 %) |
| Metopimazine | 3513 (10.8 %) | 3129 (11.9 %) | 26 (13.4 %) | 81 (5.2 %) | 277 (6.4 %) |
| Anti-D (rh) immunoglobulin | 2701 (8.3 %) | 2428 (9.2 %) | 11 (5.7 %) | 60 (3.8 %) | 202 (4.7 %) |
| Homeopathy | 2540 (7.8 %) | 2463 (9.4 %) | 11 (5.7 %) | 21 (1.3 %) | 45 (1.0 %) |
| Other drugs for acid related disorders | 2399 (7.4 %) | 2282 (8.7 %) | 13 (6.7 %) | 34 (2.2 %) | 70 (1.6 %) |
| Ibuprofen | 2367 (7.3 %) | 1522 (5.8 %) | 11 (5.7 %) | 122 (7.8 %) | 712 (16.4 %) |
| Salbutamol | 2352 (7.3 %) | 2194 (8.3 %) | 16 (8.2 %) | 43 (2.8 %) | 99 (2.3 %) |
| Amoxicillin and enzyme inhibitor | 2231 (6.9 %) | 1952 (7.4 %) | 18 (9.3 %) | 103 (6.6 %) | 158 (3.6 %) |
| Prednisolone | 2143 (6.6 %) | 1918 (7.3 %) | 11 (5.7 %) | 58 (3.7 %) | 156 (3.6 %) |
| Macrogol | 2129 (6.6 %) | 2038 (7.7 %) | 14 (7.2 %) | 30 (1.9 %) | 47 (1.1 %) |
| Levonorgestrel and estrogen | 2095 (6.5 %) | 979 (3.7 %) | 6 (3.1 %) | 138 (8.8 %) | 972 (22.4 %) |
| Various nasal preparations | 2092 (6.5 %) | 2014 (7.7 %) | 16 (8.2 %) | 20 (1.3 %) | 42 (1.0 %) |
| Povidone-iodine | 2086 (6.4 %) | 1327 (5.0 %) | 10 (5.2 %) | 191 (12.2 %) | 558 (12.9 %) |
| Chlorhexidine in combination | 2072 (6.4 %) | 1938 (7.4 %) | 10 (5.2 %) | 39 (2.5 %) | 85 (2.0 %) |
| Progesterone | 1989 (6.1 %) | 1764 (6.7 %) | 14 (7.2 %) | 135 (8.6 %) | 76 (1.8 %) |
| Soft paraffin and fat products | 1841 (5.7 %) | 1711 (6.5 %) | 12 (6.2 %) | 19 (1.2 %) | 99 (2.3 %) |
| Bromocriptine | 1828 (5.6 %) | 1792 (6.8 %) | 14 (7.2 %) | 11 (0.7 %) | 11 (0.3 %) |
| Fenticonazole | 1752 (5.4 %) | 1654 (6.3 %) | 7 (3.6 %) | 13 (0.8 %) | 78 (1.8 %) |
| Betamethasone | 1736 (5.4 %) | 1594 (6.1 %) | 12 (6.2 %) | 38 (2.4 %) | 92 (2.1 %) |
| Chlorhexidine | 1523 (4.7 %) | 1417 (5.4 %) | 8 (4.1 %) | 26 (1.7 %) | 72 (1.7 %) |
| Levonorgestrel | 1477 (4.6 %) | 1363 (5.2 %) | 7 (3.6 %) | 15 (1.0 %) | 92 (2.1 %) |
| Cefixime | 1467 (4.5 %) | 1387 (5.3 %) | 18 (9.3 %) | 20 (1.3 %) | 42 (1.0 %) |
| Stomatological preparations (antiinfectives and antiseptics for local oral treatment) | 1457 (4.5 %) | 1323 (5.0 %) | 8 (4.1 %) | 37 (2.4 %) | 89 (2.1 %) |
| Nystatin in combination | 1401 (4.3 %) | 1251 (4.8 %) | 8 (4.1 %) | 35 (2.2 %) | 107 (2.5 %) |
| Enoxaparin | 1371 (4.2 %) | 1319 (5.0 %) | 9 (4.6 %) | 31 (2.0 %) | 12 (0.3 %) |
| Dextromethorphan | 1316 (4.1 %) | 1224 (4.7 %) | 6 (3.1 %) | 20 (1.3 %) | 66 (1.5 %) |
| Fosfomycin | 1167 (3.6 %) | 1051 (4.0 %) | 7 (3.6 %) | 39 (2.5 %) | 70 (1.6 %) |
| Esomeprazole | 1144 (3.5 %) | 1025 (3.9 %) | 9 (4.6 %) | 32 (2.0 %) | 78 (1.8 %) |
| Ciclopirox | 1134 (3.5 %) | 1062 (4.0 %) | 6 (3.1 %) | 16 (1.0 %) | 50 (1.2 %) |
| Metronidazole | 1100 (3.4 %) | 1017 (3.9 %) | 6 (3.1 %) | 12 (0.8 %) | 65 (1.5 %) |
| Codeine in combination | 1089 (3.4 %) | 840 (3.2 %) | 8 (4.1 %) | 54 (3.5 %) | 187 (4.3 %) |
| Acetylsalicylic acid | 1045 (3.2 %) | 937 (3.6 %) | 13 (6.7 %) | 45 (2.9 %) | 50 (1.2 %) |
| Diclofenac | 1043 (3.2 %) | 862 (3.3 %) | 7 (3.6 %) | 45 (2.9 %) | 129 (3.0 %) |
| Whooping cough, tetanus, diphtheria and polio | 1020 (3.1 %) | 978 (3.7 %) | 0 (0%) | 10 (0.6 %) | 32 (0.7 %) |
| Ketoprofen | 1014 (3.1 %) | 718 (2.7 %) | 3 (1.5 %) | 71 (4.5 %) | 222 (5.1 %) |
| Acid fusidic | 1006 (3.1 %) | 908 (3.4 %) | 3 (1.5 %) | 28 (1.8 %) | 67 (1.5 %) |
| Levothyroxine sodium | 959 (3.0 %) | 814 (3.1 %) | 10 (5.2 %) | 56 (3.6 %) | 79 (1.8 %) |

**Note:** The sample size can vary given that some pregnancies did not use medication. A pregnancy can be counted more than once if a woman used more than one medications.

**Table I. Top 50 medication use in the year following the pregnancy (yes vs. no): stratified by pregnancy outcomes**

| **Top 50 medication use in the year following the pregnancy (yes vs. no)** | **All pregnancies 2010 - 2013 (n=34475)** | **Livebirths (n=26179)** | **Stillbirths (n=197)** | **Spontaneous abortions (n=1968)** | **Planned abortions (n=6131)** |
| --- | --- | --- | --- | --- | --- |
| Acetaminophen/paracetamol | 26428 (76.7 %) | 20509 (78.3 %) | 157 (79.7 %) | 1547 (78.6 %) | 4215 (68.7 %) |
| Ferrous sulfate | 11532 (33.5 %) | 9845 (37.6 %) | 80 (40.6 %) | 678 (34.5 %) | 929 (15.2 %) |
| Levonorgestrel and estrogen | 10866 (31.5 %) | 8178 (31.2 %) | 44 (22.3 %) | 428 (21.7 %) | 2216 (36.1 %) |
| Phloroglucinol | 10823 (31.4 %) | 7772 (29.7 %) | 91 (46.2 %) | 954 (48.5 %) | 2006 (32.7 %) |
| Ibuprofen | 10471 (30.4 %) | 7833 (29.9 %) | 71 (36.0 %) | 516 (26.2 %) | 2051 (33.5 %) |
| Amoxicillin | 8679 (25.2 %) | 6617 (25.3 %) | 57 (28.9 %) | 533 (27.1 %) | 1472 (24.0 %) |
| Prednisolone | 6052 (17.6 %) | 4549 (17.4 %) | 30 (15.2 %) | 354 (18.0 %) | 1119 (18.3 %) |
| Diclofenac | 5004 (14.5 %) | 3655 (14.0 %) | 36 (18.3 %) | 284 (14.4 %) | 1029 (16.8 %) |
| Amoxicillin and enzyme inhibitor | 4908 (14.2 %) | 3611 (13.8 %) | 34 (17.3 %) | 299 (15.2 %) | 964 (15.7 %) |
| Colecalciferol | 4823 (14.0 %) | 3671 (14.0 %) | 41 (20.8 %) | 461 (23.4 %) | 650 (10.6 %) |
| Ketoprofen | 4816 (14.0 %) | 3552 (13.6 %) | 28 (14.2 %) | 268 (13.6 %) | 968 (15.8 %) |
| Tixocortol | 4496 (13.0 %) | 3538 (13.5 %) | 22 (11.2 %) | 294 (14.9 %) | 642 (10.5 %) |
| Bromocriptine | 4228 (12.3 %) | 4066 (15.5 %) | 40 (20.3 %) | 66 (3.4 %) | 56 (0.9 %) |
| Levonorgestrel | 4213 (12.2 %) | 3891 (14.9 %) | 20 (10.2 %) | 81 (4.1 %) | 221 (3.6 %) |
| Whooping cough, tetanus, diphtheria and polio | 4029 (11.7 %) | 3618 (13.8 %) | 17 (8.6 %) | 114 (5.8 %) | 280 (4.6 %) |
| Intrauterine devices with progestin | 3702 (10.7 %) | 3204 (12.2 %) | 4 (2.0 %) | 50 (2.5 %) | 444 (7.2 %) |
| Helicidine | 3698 (10.7 %) | 2794 (10.7 %) | 24 (12.2 %) | 273 (13.9 %) | 607 (9.9 %) |
| Betamethasone | 3490 (10.1 %) | 2658 (10.2 %) | 22 (11.2 %) | 193 (9.8 %) | 617 (10.1 %) |
| Chlorhexidine in combination | 3467 (10.1 %) | 2739 (10.5 %) | 21 (10.7 %) | 187 (9.5 %) | 520 (8.5 %) |
| Povidone-iodine | 3445 (10.0 %) | 2322 (8.9 %) | 20 (10.2 %) | 309 (15.7 %) | 794 (13.0 %) |
| Domperidone | 3394 (9.8 %) | 2434 (9.3 %) | 17 (8.6 %) | 227 (11.5 %) | 716 (11.7 %) |
| Omeprazole | 3039 (8.8 %) | 2179 (8.3 %) | 27 (13.7 %) | 243 (12.3 %) | 590 (9.6 %) |
| Codeine in combination | 3016 (8.7 %) | 2038 (7.8 %) | 20 (10.2 %) | 194 (9.9 %) | 764 (12.5 %) |
| Stomatological preparations (antiinfectives and antiseptics for local oral treatment) | 3005 (8.7 %) | 2228 (8.5 %) | 20 (10.2 %) | 152 (7.7 %) | 605 (9.9 %) |
| Econazole | 2977 (8.6 %) | 2106 (8.0 %) | 28 (14.2 %) | 239 (12.1 %) | 604 (9.9 %) |
| Desloratadine | 2943 (8.5 %) | 2136 (8.2 %) | 13 (6.6 %) | 180 (9.1 %) | 614 (10.0 %) |
| Chlorhexidine | 2697 (7.8 %) | 2052 (7.8 %) | 10 (5.1 %) | 144 (7.3 %) | 491 (8.0 %) |
| Folic acid | 2613 (7.6 %) | 1347 (5.1 %) | 85 (43.1 %) | 731 (37.1 %) | 450 (7.3 %) |
| Pholcodine | 2536 (7.4 %) | 1888 (7.2 %) | 12 (6.1 %) | 139 (7.1 %) | 497 (8.1 %) |
| Other drugs for acid related disorders | 2517 (7.3 %) | 1930 (7.4 %) | 15 (7.6 %) | 170 (8.6 %) | 402 (6.6 %) |
| Metopimazine | 2428 (7.0 %) | 1633 (6.2 %) | 16 (8.1 %) | 188 (9.6 %) | 591 (9.6 %) |
| Sertaconazole | 2415 (7.0 %) | 1649 (6.3 %) | 28 (14.2 %) | 231 (11.7 %) | 507 (8.3 %) |
| Enoxaparin | 2368 (6.9 %) | 2096 (8.0 %) | 21 (10.7 %) | 134 (6.8 %) | 117 (1.9 %) |
| Cefpodoxime | 2365 (6.9 %) | 1799 (6.9 %) | 14 (7.1 %) | 122 (6.2 %) | 430 (7.0 %) |
| Soft paraffin and fat products | 2363 (6.9 %) | 1787 (6.8 %) | 13 (6.6 %) | 139 (7.1 %) | 424 (6.9 %) |
| Prednisolone with association | 2336 (6.8 %) | 1647 (6.3 %) | 12 (6.1 %) | 143 (7.3 %) | 534 (8.7 %) |
| Acid tiaprofenic | 2202 (6.4 %) | 1647 (6.3 %) | 14 (7.1 %) | 80 (4.1 %) | 461 (7.5 %) |
| Salbutamol | 2186 (6.3 %) | 1566 (6.0 %) | 15 (7.6 %) | 153 (7.8 %) | 452 (7.4 %) |
| Oxomemazine | 2144 (6.2 %) | 1511 (5.8 %) | 8 (4.1 %) | 134 (6.8 %) | 491 (8.0 %) |
| Racecadotril | 2041 (5.9 %) | 1601 (6.1 %) | 13 (6.6 %) | 86 (4.4 %) | 341 (5.6 %) |
| Acid fusidic | 2033 (5.9 %) | 1593 (6.1 %) | 13 (6.6 %) | 127 (6.5 %) | 300 (4.9 %) |
| Lidocaine combination | 2024 (5.9 %) | 1671 (6.4 %) | 8 (4.1 %) | 44 (2.2 %) | 301 (4.9 %) |
| Etonogestrel | 1943 (5.6 %) | 1473 (5.6 %) | 7 (3.6 %) | 50 (2.5 %) | 413 (6.7 %) |
| Tramadol in combination | 1934 (5.6 %) | 1260 (4.8 %) | 7 (3.6 %) | 147 (7.5 %) | 520 (8.5 %) |
| Esomeprazole | 1917 (5.6 %) | 1387 (5.3 %) | 22 (11.2 %) | 141 (7.2 %) | 367 (6.0 %) |
| Dextromethorphan | 1888 (5.5 %) | 1453 (5.6 %) | 15 (7.6 %) | 100 (5.1 %) | 320 (5.2 %) |
| Paracetamol combinations with psycholeptics | 1860 (5.4 %) | 1107 (4.2 %) | 11 (5.6 %) | 104 (5.3 %) | 638 (10.4 %) |
| Alginic acid in combination with antacids | 1853 (5.4 %) | 1234 (4.7 %) | 20 (10.2 %) | 251 (12.8 %) | 348 (5.7 %) |
| Loperamide | 1835 (5.3 %) | 1365 (5.2 %) | 6 (3.0 %) | 110 (5.6 %) | 354 (5.8 %) |
| Dexamethasone and antiinfectives | 1782 (5.2 %) | 1353 (5.2 %) | 10 (5.1 %) | 92 (4.7 %) | 327 (5.3 %) |

**Note:** The sample size can vary given that some pregnancies did not use medication. A pregnancy can be counted more than once if a woman used more than one medications.

**Table J. Top 50 medication use during the first trimester of pregnancy (yes vs. no): stratified by pregnancy outcomes**

| **Top 50 medication use during the first trimester of pregnancy (yes vs. no**) | **All pregnancies 2010 - 2013 (n=27554)** | **Livebirths (n=21524)** | **Stillbirths (n=169)** | **Spontaneous abortions (n=1553)** | **Planned abortions (n=4308)** |
| --- | --- | --- | --- | --- | --- |
| Acetaminophen/paracetamol | 13318 (48.3 %) | 10836 (50.3 %) | 88 (52.1 %) | 811 (52.2 %) | 1583 (36.7 %) |
| Phloroglucinol | 8103 (29.4 %) | 6661 (30.9 %) | 62 (36.7 %) | 491 (31.6 %) | 889 (20.6 %) |
| Folic acid | 7060 (25.6 %) | 6461 (30.0 %) | 44 (26.0 %) | 373 (24.0 %) | 182 (4.2 %) |
| Metoclopramide | 3949 (14.3 %) | 3579 (16.6 %) | 29 (17.2 %) | 91 (5.9 %) | 250 (5.8 %) |
| Ferrous sulfate | 3691 (13.4 %) | 3288 (15.3 %) | 29 (17.2 %) | 194 (12.5 %) | 180 (4.2 %) |
| Domperidone | 3506 (12.7 %) | 3084 (14.3 %) | 26 (15.4 %) | 92 (5.9 %) | 304 (7.1 %) |
| Amoxicillin | 3390 (12.3 %) | 2947 (13.7 %) | 28 (16.6 %) | 142 (9.1 %) | 273 (6.3 %) |
| Metopimazine | 2865 (10.4 %) | 2487 (11.6 %) | 21 (12.4 %) | 80 (5.2 %) | 277 (6.4 %) |
| Helicidine | 2592 (9.4 %) | 2372 (11.0 %) | 15 (8.9 %) | 79 (5.1 %) | 126 (2.9 %) |
| Tixocortol | 2198 (8.0 %) | 1998 (9.3 %) | 15 (8.9 %) | 71 (4.6 %) | 114 (2.6 %) |
| Alginic acid in combination with antacids | 2140 (7.8 %) | 2004 (9.3 %) | 19 (11.2 %) | 51 (3.3 %) | 66 (1.5 %) |
| Progesterone | 1856 (6.7 %) | 1634 (7.6 %) | 14 (8.3 %) | 133 (8.6 %) | 75 (1.7 %) |
| Sertaconazole | 1559 (5.7 %) | 1392 (6.5 %) | 10 (5.9 %) | 43 (2.8 %) | 114 (2.6 %) |
| Econazole | 1546 (5.6 %) | 1367 (6.4 %) | 10 (5.9 %) | 52 (3.3 %) | 117 (2.7 %) |
| Colecalciferol | 1405 (5.1 %) | 1255 (5.8 %) | 8 (4.7 %) | 37 (2.4 %) | 105 (2.4 %) |
| Ibuprofen | 1392 (5.1 %) | 560 (2.6 %) | 7 (4.1 %) | 119 (7.7 %) | 706 (16.4 %) |
| Levonorgestrel and estrogen | 1333 (4.8 %) | 229 (1.1 %) | 1 (0.6 %) | 138 (8.9 %) | 965 (22.4 %) |
| Other drugs for acid related disorders | 1179 (4.3 %) | 1069 (5.0 %) | 9 (5.3 %) | 33 (2.1 %) | 68 (1.6 %) |
| Omeprazole | 1158 (4.2 %) | 994 (4.6 %) | 14 (8.3 %) | 48 (3.1 %) | 102 (2.4 %) |
| Povidone-iodine | 1054 (3.8 %) | 310 (1.4 %) | 2 (1.2 %) | 186 (12.0 %) | 556 (12.9 %) |
| Prednisolone | 1038 (3.8 %) | 823 (3.8 %) | 7 (4.1 %) | 58 (3.7 %) | 150 (3.5 %) |
| Amoxicillin and enzyme inhibitor | 1030 (3.7 %) | 777 (3.6 %) | 4 (2.4 %) | 98 (6.3 %) | 151 (3.5 %) |
| Various nasal preparations | 979 (3.6 %) | 911 (4.2 %) | 8 (4.7 %) | 20 (1.3 %) | 40 (0.9 %) |
| Macrogol | 964 (3.5 %) | 884 (4.1 %) | 8 (4.7 %) | 28 (1.8 %) | 44 (1.0 %) |
| Salbutamol | 878 (3.2 %) | 735 (3.4 %) | 4 (2.4 %) | 42 (2.7 %) | 97 (2.3 %) |
| Acetylsalicylic acid | 852 (3.1 %) | 750 (3.5 %) | 10 (5.9 %) | 45 (2.9 %) | 47 (1.1 %) |
| Homeopathy | 831 (3.0 %) | 762 (3.5 %) | 5 (3.0 %) | 21 (1.4 %) | 43 (1.0 %) |
| Levothyroxine sodium | 797 (2.9 %) | 655 (3.0 %) | 7 (4.1 %) | 56 (3.6 %) | 79 (1.8 %) |
| Soft paraffin and fat products | 789 (2.9 %) | 667 (3.1 %) | 6 (3.6 %) | 19 (1.2 %) | 97 (2.3 %) |
| Betamethasone | 731 (2.7 %) | 599 (2.8 %) | 6 (3.6 %) | 36 (2.3 %) | 90 (2.1 %) |
| Stomatological preparations (antiinfectives and antiseptics for local oral treatment) | 691 (2.5 %) | 566 (2.6 %) | 4 (2.4 %) | 36 (2.3 %) | 85 (2.0 %) |
| Diclofenac | 666 (2.4 %) | 493 (2.3 %) | 3 (1.8 %) | 45 (2.9 %) | 125 (2.9 %) |
| Chlorhexidine in combination | 661 (2.4 %) | 534 (2.5 %) | 8 (4.7 %) | 36 (2.3 %) | 83 (1.9 %) |
| Chlorhexidine | 633 (2.3 %) | 535 (2.5 %) | 3 (1.8 %) | 25 (1.6 %) | 70 (1.6 %) |
| Codeine in combination | 613 (2.2 %) | 373 (1.7 %) | 3 (1.8 %) | 53 (3.4 %) | 184 (4.3 %) |
| Nystatin in combination | 595 (2.2 %) | 454 (2.1 %) | 3 (1.8 %) | 33 (2.1 %) | 105 (2.4 %) |
| Fenticonazole | 587 (2.1 %) | 498 (2.3 %) | 1 (0.6 %) | 11 (0.7 %) | 77 (1.8 %) |
| Cefixime | 579 (2.1 %) | 513 (2.4 %) | 7 (4.1 %) | 19 (1.2 %) | 40 (0.9 %) |
| Dextromethorphan | 570 (2.1 %) | 485 (2.3 %) | 3 (1.8 %) | 20 (1.3 %) | 62 (1.4 %) |
| Fosfomycin | 559 (2.0 %) | 449 (2.1 %) | 4 (2.4 %) | 38 (2.4 %) | 68 (1.6 %) |
| Anti-D (rh) immunoglobulin | 539 (2.0 %) | 275 (1.3 %) | 3 (1.8 %) | 59 (3.8 %) | 202 (4.7 %) |
| Desloratadine | 524 (1.9 %) | 394 (1.8 %) | 3 (1.8 %) | 28 (1.8 %) | 99 (2.3 %) |
| Ketoprofen | 519 (1.9 %) | 233 (1.1 %) | 0 (0%) | 68 (4.4 %) | 218 (5.1 %) |
| Mometasone | 483 (1.8 %) | 413 (1.9 %) | 3 (1.8 %) | 22 (1.4 %) | 45 (1.0 %) |
| Paracetamol combinations with psycholeptics | 483 (1.8 %) | 104 (0.5 %) | 1 (0.6 %) | 47 (3.0 %) | 331 (7.7 %) |
| Ciclopirox | 467 (1.7 %) | 396 (1.8 %) | 5 (3.0 %) | 16 (1.0 %) | 50 (1.2 %) |
| Acid fusidic | 449 (1.6 %) | 356 (1.7 %) | 0 (0%) | 28 (1.8 %) | 65 (1.5 %) |
| Beclometasone | 444 (1.6 %) | 372 (1.7 %) | 1 (0.6 %) | 20 (1.3 %) | 51 (1.2 %) |
| Tuaminoheptane | 434 (1.6 %) | 334 (1.6 %) | 4 (2.4 %) | 28 (1.8 %) | 68 (1.6 %) |
| Esomeprazole | 429 (1.6 %) | 320 (1.5 %) | 2 (1.2 %) | 32 (2.1 %) | 75 (1.7 %) |

**Note:** The sample size can vary given that some pregnancies did not use medication. A pregnancy can be counted more than once if a woman used more than one medications.

**Table K. Top 50 medication use during the second trimester of pregnancy (yes vs. no): stratified by pregnancy outcomes**

| **Top 50 medication use during the second trimester of pregnancy (yes vs. no**) | **All pregnancies 2010 - 2013 (n=22416)** | **Livebirths (n=22180)** | **Stillbirths (n=161)** | **Spontaneous abortions (n=43)** | **Planned abortions (n=32)** |
| --- | --- | --- | --- | --- | --- |
| Ferrous sulfate | 9700 (43.3 %) | 9629 (43.4 %) | 54 (33.5 %) | 14 (32.6 %) | 3 (9.4 %) |
| Acetaminophen/paracetamol | 9601 (42.8 %) | 9490 (42.8 %) | 70 (43.5 %) | 22 (51.2 %) | 19 (59.4 %) |
| Colecalciferol | 7023 (31.3 %) | 6995 (31.5 %) | 27 (16.8 %) | 1 (2.3 %) | 0 (0%) |
| Phloroglucinol | 6514 (29.1 %) | 6433 (29.0 %) | 52 (32.3 %) | 18 (41.9 %) | 11 (34.4 %) |
| Amoxicillin | 2813 (12.5 %) | 2785 (12.6 %) | 19 (11.8 %) | 8 (18.6 %) | 1 (3.1 %) |
| Alginic acid in combination with antacids | 2810 (12.5 %) | 2793 (12.6 %) | 15 (9.3 %) | 1 (2.3 %) | 1 (3.1 %) |
| Helicidine | 2179 (9.7 %) | 2167 (9.8 %) | 12 (7.5 %) | 0 (0%) | 0 (0%) |
| Sertaconazole | 1828 (8.2 %) | 1815 (8.2 %) | 11 (6.8 %) | 2 (4.7 %) | 0 (0%) |
| Tixocortol | 1800 (8.0 %) | 1791 (8.1 %) | 8 (5.0 %) | 0 (0%) | 1 (3.1 %) |
| Econazole | 1775 (7.9 %) | 1767 (8.0 %) | 7 (4.3 %) | 1 (2.3 %) | 0 (0%) |
| Omeprazole | 1404 (6.3 %) | 1395 (6.3 %) | 7 (4.3 %) | 2 (4.7 %) | 0 (0%) |
| Anti-D (rh) immunoglobulin | 1353 (6.0 %) | 1346 (6.1 %) | 6 (3.7 %) | 1 (2.3 %) | 0 (0%) |
| Salbutamol | 985 (4.4 %) | 977 (4.4 %) | 8 (5.0 %) | 0 (0%) | 0 (0%) |
| Folic acid | 944 (4.2 %) | 927 (4.2 %) | 10 (6.2 %) | 5 (11.6 %) | 2 (6.3 %) |
| Homeopathy | 870 (3.9 %) | 862 (3.9 %) | 6 (3.7 %) | 0 (0%) | 2 (6.3 %) |
| Various nasal preparations | 811 (3.6 %) | 801 (3.6 %) | 9 (5.6 %) | 0 (0%) | 1 (3.1 %) |
| Other drugs for acid related disorders | 799 (3.6 %) | 795 (3.6 %) | 3 (1.9 %) | 1 (2.3 %) | 0 (0%) |
| Macrogol | 773 (3.4 %) | 768 (3.5 %) | 3 (1.9 %) | 1 (2.3 %) | 1 (3.1 %) |
| Soft paraffin and fat products | 754 (3.4 %) | 744 (3.4 %) | 10 (6.2 %) | 0 (0%) | 0 (0%) |
| Prednisolone | 748 (3.3 %) | 744 (3.4 %) | 4 (2.5 %) | 0 (0%) | 0 (0%) |
| Levothyroxine sodium | 718 (3.2 %) | 709 (3.2 %) | 9 (5.6 %) | 0 (0%) | 0 (0%) |
| Amoxicillin and enzyme inhibitor | 709 (3.2 %) | 694 (3.1 %) | 11 (6.8 %) | 4 (9.3 %) | 0 (0%) |
| Fenticonazole | 704 (3.1 %) | 697 (3.1 %) | 6 (3.7 %) | 1 (2.3 %) | 0 (0%) |
| Domperidone | 629 (2.8 %) | 626 (2.8 %) | 3 (1.9 %) | 0 (0%) | 0 (0%) |
| Metoclopramide | 585 (2.6 %) | 581 (2.6 %) | 3 (1.9 %) | 1 (2.3 %) | 0 (0%) |
| Betamethasone | 576 (2.6 %) | 570 (2.6 %) | 5 (3.1 %) | 1 (2.3 %) | 0 (0%) |
| Metopimazine | 555 (2.5 %) | 550 (2.5 %) | 5 (3.1 %) | 0 (0%) | 0 (0%) |
| Chlorhexidine | 533 (2.4 %) | 528 (2.4 %) | 4 (2.5 %) | 1 (2.3 %) | 0 (0%) |
| Cefixime | 533 (2.4 %) | 521 (2.3 %) | 8 (5.0 %) | 2 (4.7 %) | 2 (6.3 %) |
| Acetylsalicylic acid | 528 (2.4 %) | 515 (2.3 %) | 10 (6.2 %) | 1 (2.3 %) | 2 (6.3 %) |
| Dextromethorphan | 512 (2.3 %) | 507 (2.3 %) | 2 (1.2 %) | 1 (2.3 %) | 2 (6.3 %) |
| Stomatological preparations (antiinfectives and antiseptics for local oral treatment) | 511 (2.3 %) | 504 (2.3 %) | 5 (3.1 %) | 1 (2.3 %) | 1 (3.1 %) |
| Chlorhexidine in combination | 507 (2.3 %) | 500 (2.3 %) | 5 (3.1 %) | 2 (4.7 %) | 0 (0%) |
| Nystatin in combination | 451 (2.0 %) | 445 (2.0 %) | 2 (1.2 %) | 3 (7.0 %) | 1 (3.1 %) |
| Ciclopirox | 441 (2.0 %) | 440 (2.0 %) | 1 (0.6 %) | 0 (0%) | 0 (0%) |
| Fosfomycin | 377 (1.7 %) | 375 (1.7 %) | 0 (0%) | 1 (2.3 %) | 1 (3.1 %) |
| Mometasone | 365 (1.6 %) | 361 (1.6 %) | 4 (2.5 %) | 0 (0%) | 0 (0%) |
| Beclometasone | 346 (1.5 %) | 342 (1.5 %) | 4 (2.5 %) | 0 (0%) | 0 (0%) |
| magnesium | 328 (1.5 %) | 325 (1.5 %) | 2 (1.2 %) | 1 (2.3 %) | 0 (0%) |
| Acid fusidic | 326 (1.5 %) | 323 (1.5 %) | 3 (1.9 %) | 0 (0%) | 0 (0%) |
| Esomeprazole | 323 (1.4 %) | 318 (1.4 %) | 5 (3.1 %) | 0 (0%) | 0 (0%) |
| Metronidazole | 321 (1.4 %) | 319 (1.4 %) | 2 (1.2 %) | 0 (0%) | 0 (0%) |
| Codeine in combination | 307 (1.4 %) | 303 (1.4 %) | 3 (1.9 %) | 0 (0%) | 1 (3.1 %) |
| Cetirizine | 302 (1.3 %) | 299 (1.3 %) | 3 (1.9 %) | 0 (0%) | 0 (0%) |
| Spiramycin | 302 (1.3 %) | 297 (1.3 %) | 4 (2.5 %) | 1 (2.3 %) | 0 (0%) |
| Povidone-iodine | 287 (1.3 %) | 280 (1.3 %) | 2 (1.2 %) | 3 (7.0 %) | 2 (6.3 %) |
| Desloratadine | 286 (1.3 %) | 283 (1.3 %) | 3 (1.9 %) | 0 (0%) | 0 (0%) |
| Hydroxyzine | 276 (1.2 %) | 264 (1.2 %) | 10 (6.2 %) | 2 (4.7 %) | 0 (0%) |
| Lactulose | 275 (1.2 %) | 268 (1.2 %) | 6 (3.7 %) | 1 (2.3 %) | 0 (0%) |
| Erythromycin | 264 (1.2 %) | 264 (1.2 %) | 0 (0%) | 0 (0%) | 0 (0%) |

**Note:** The sample size can vary given that some pregnancies did not use medication. A pregnancy can be counted more than once if a woman used more than one medications.

**Table L.** Top 50 medication use during the third trimester of pregnancy (yes vs. no): stratified by pregnancy outcomes

| **Top 50 medication use during the third trimester of pregnancy (yes vs. no**) | **All pregnancies 2010 - 2013 (n=23932)** | **Livebirths (n=23861)** | **Stillbirths (n=71)** | **Spontaneous abortions (n=.)** | **Planned abortions (n=.)** |
| --- | --- | --- | --- | --- | --- |
| Ferrous sulfate | 14381 (60.1 %) | 14341 (60.1 %) | 40 (56.3 %) | 0 (0%) | 0 (0%) |
| Acetaminophen/paracetamol | 11227 (46.9 %) | 11196 (46.9 %) | 31 (43.7 %) | 0 (0%) | 0 (0%) |
| Phloroglucinol | 6730 (28.1 %) | 6715 (28.1 %) | 15 (21.1 %) | 0 (0%) | 0 (0%) |
| Colecalciferol | 5829 (24.4 %) | 5821 (24.4 %) | 8 (11.3 %) | 0 (0%) | 0 (0%) |
| Alginic acid in combination with antacids | 3089 (12.9 %) | 3083 (12.9 %) | 6 (8.5 %) | 0 (0%) | 0 (0%) |
| Amoxicillin | 2392 (10.0 %) | 2387 (10.0 %) | 5 (7.0 %) | 0 (0%) | 0 (0%) |
| Omeprazole | 2152 (9.0 %) | 2147 (9.0 %) | 5 (7.0 %) | 0 (0%) | 0 (0%) |
| Sertaconazole | 2127 (8.9 %) | 2124 (8.9 %) | 3 (4.2 %) | 0 (0%) | 0 (0%) |
| Econazole | 1930 (8.1 %) | 1927 (8.1 %) | 3 (4.2 %) | 0 (0%) | 0 (0%) |
| Bromocriptine | 1771 (7.4 %) | 1766 (7.4 %) | 5 (7.0 %) | 0 (0%) | 0 (0%) |
| Helicidine | 1522 (6.4 %) | 1519 (6.4 %) | 3 (4.2 %) | 0 (0%) | 0 (0%) |
| Folic acid | 1361 (5.7 %) | 1358 (5.7 %) | 3 (4.2 %) | 0 (0%) | 0 (0%) |
| Levonorgestrel | 1332 (5.6 %) | 1330 (5.6 %) | 2 (2.8 %) | 0 (0%) | 0 (0%) |
| Tixocortol | 1273 (5.3 %) | 1269 (5.3 %) | 4 (5.6 %) | 0 (0%) | 0 (0%) |
| Homeopathy | 1240 (5.2 %) | 1239 (5.2 %) | 1 (1.4 %) | 0 (0%) | 0 (0%) |
| Enoxaparin | 1195 (5.0 %) | 1189 (5.0 %) | 6 (8.5 %) | 0 (0%) | 0 (0%) |
| Salbutamol | 1083 (4.5 %) | 1077 (4.5 %) | 6 (8.5 %) | 0 (0%) | 0 (0%) |
| Chlorhexidine in combination | 1082 (4.5 %) | 1082 (4.5 %) | 0 (0%) | 0 (0%) | 0 (0%) |
| Anti-D (rh) immunoglobulin | 1013 (4.2 %) | 1011 (4.2 %) | 2 (2.8 %) | 0 (0%) | 0 (0%) |
| Whooping cough, tetanus, diphtheria and polio | 881 (3.7 %) | 881 (3.7 %) | 0 (0%) | 0 (0%) | 0 (0%) |
| Ibuprofen | 844 (3.5 %) | 841 (3.5 %) | 3 (4.2 %) | 0 (0%) | 0 (0%) |
| Povidone-iodine | 787 (3.3 %) | 781 (3.3 %) | 6 (8.5 %) | 0 (0%) | 0 (0%) |
| Levothyroxine sodium | 743 (3.1 %) | 738 (3.1 %) | 5 (7.0 %) | 0 (0%) | 0 (0%) |
| Levonorgestrel and estrogen | 738 (3.1 %) | 735 (3.1 %) | 3 (4.2 %) | 0 (0%) | 0 (0%) |
| Soft paraffin and fat products | 733 (3.1 %) | 733 (3.1 %) | 0 (0%) | 0 (0%) | 0 (0%) |
| Fenticonazole | 709 (3.0 %) | 709 (3.0 %) | 0 (0%) | 0 (0%) | 0 (0%) |
| Nicardipine | 700 (2.9 %) | 696 (2.9 %) | 4 (5.6 %) | 0 (0%) | 0 (0%) |
| Amoxicillin and enzyme inhibitor | 676 (2.8 %) | 672 (2.8 %) | 4 (5.6 %) | 0 (0%) | 0 (0%) |
| Macrogol | 654 (2.7 %) | 651 (2.7 %) | 3 (4.2 %) | 0 (0%) | 0 (0%) |
| Esomeprazole | 630 (2.6 %) | 627 (2.6 %) | 3 (4.2 %) | 0 (0%) | 0 (0%) |
| calcium carbonate | 586 (2.4 %) | 584 (2.4 %) | 2 (2.8 %) | 0 (0%) | 0 (0%) |
| Other preparations in combination | 584 (2.4 %) | 583 (2.4 %) | 1 (1.4 %) | 0 (0%) | 0 (0%) |
| Betamethasone | 583 (2.4 %) | 582 (2.4 %) | 1 (1.4 %) | 0 (0%) | 0 (0%) |
| Prednisolone | 558 (2.3 %) | 558 (2.3 %) | 0 (0%) | 0 (0%) | 0 (0%) |
| Various nasal preparations | 529 (2.2 %) | 528 (2.2 %) | 1 (1.4 %) | 0 (0%) | 0 (0%) |
| Other drugs for acid related disorders | 526 (2.2 %) | 525 (2.2 %) | 1 (1.4 %) | 0 (0%) | 0 (0%) |
| Carbonic anhydrase inhibitor drugs | 514 (2.1 %) | 512 (2.1 %) | 2 (2.8 %) | 0 (0%) | 0 (0%) |
| Calcium in combination with vitamin D and / or other drugs | 513 (2.1 %) | 512 (2.1 %) | 1 (1.4 %) | 0 (0%) | 0 (0%) |
| Cefixime | 496 (2.1 %) | 493 (2.1 %) | 3 (4.2 %) | 0 (0%) | 0 (0%) |
| Nystatin in combination | 489 (2.0 %) | 486 (2.0 %) | 3 (4.2 %) | 0 (0%) | 0 (0%) |
| Chlorhexidine | 485 (2.0 %) | 484 (2.0 %) | 1 (1.4 %) | 0 (0%) | 0 (0%) |
| Ferrous fumarate | 471 (2.0 %) | 471 (2.0 %) | 0 (0%) | 0 (0%) | 0 (0%) |
| Nifedipine | 459 (1.9 %) | 458 (1.9 %) | 1 (1.4 %) | 0 (0%) | 0 (0%) |
| Metronidazole | 452 (1.9 %) | 451 (1.9 %) | 1 (1.4 %) | 0 (0%) | 0 (0%) |
| Ketoprofen | 413 (1.7 %) | 411 (1.7 %) | 2 (2.8 %) | 0 (0%) | 0 (0%) |
| Etonogestrel | 389 (1.6 %) | 389 (1.6 %) | 0 (0%) | 0 (0%) | 0 (0%) |
| Hydroxyzine | 376 (1.6 %) | 372 (1.6 %) | 4 (5.6 %) | 0 (0%) | 0 (0%) |
| Insulin aspart | 362 (1.5 %) | 362 (1.5 %) | 0 (0%) | 0 (0%) | 0 (0%) |
| Ranitidine | 358 (1.5 %) | 357 (1.5 %) | 1 (1.4 %) | 0 (0%) | 0 (0%) |
| Stomatological preparations (antiinfectives and antiseptics for local oral treatment) | 337 (1.4 %) | 336 (1.4 %) | 1 (1.4 %) | 0 (0%) | 0 (0%) |

**Note:** The sample size can vary given that some pregnancies did not use medication. A pregnancy can be counted more than once if a woman used more than one medications.
